# Association of Immune Checkpoint Inhibitors With Neurologic Adverse Events: A Systematic Review and Meta-analysis

**DOI:** 10.1001/jamanetworkopen.2022.7722

**Published:** 2022-04-19

**Authors:** Muhammad Zain Farooq, Sheeba Ba Aqeel, Prasanth Lingamaneni, Rayli Carolina Pichardo, Aleeza Jawed, Saad Khalid, Shristi Upadhyay Banskota, Pingfu Fu, Ankit Mangla

**Affiliations:** 1 Department of Hematology and Oncology, Moffitt Cancer Center, University of South Florida, Tampa; 2 Roswell Park Comprehensive Cancer Center, Buffalo, New York; 3 Department of Internal Medicine, John H. Stroger Jr, Hospital of Cook County, Chicago, Illinois; 4 Department of Hematology and Oncology, Henry Ford Health System, Detroit, Michigan; 5 Department of Internal Medicine, Ziauddin University Hospital, Karachi, Pakistan; 6 Department of Internal Medicine, Dow University of Health Sciences, Karachi, Pakistan; 7 Department of Hematology and Oncology, University of Nebraska Medical Center, Lincoln; 8 Department of Population and Quantitative Health Sciences, Case Western Reserve University School of Medicine, Cleveland, Ohio; 9 Division of Hematology and Oncology, Case Western Reserve University School of Medicine, Cleveland, Ohio

## Abstract

**Importance:**

Neurologic adverse events (NAEs) due to immune checkpoint inhibitors (ICIs) can be fatal but are underexplored.

**Objective:**

To compare NAEs reported in randomized clinical trials (RCTs) of US Food and Drug Administration–approved ICIs with other forms of chemotherapy and placebo.

**Data Sources:**

Bibliographic databases (Embase, Ovid, MEDLINE, and Scopus data) and trial registries (ClinicalTrials.gov) were searched from inception through March 1, 2020.

**Study Selection:**

Phase II/III RCTs evaluating the use of ICIs were eligible for inclusion. Unpublished trials were excluded from the analysis.

**Data Extraction and Synthesis:**

Two investigators independently performed screening of trials using the Preferred Reporting Items for Systematic Reviews and Meta-analyses (PRISMA) guideline. NAEs were recorded for each arm. Data were pooled using a random-effects model.

**Main Outcomes and Measures:**

The risk of NAEs with ICI use compared with any drug regimen, cytotoxic chemotherapy, and placebo.

**Results:**

A total 39 trials including 23 705 patients were analyzed (16 135 [68.0%] men, 7866 [33.1%] White). The overall risk of a NAE was lower in the ICI group (risk ratio [RR], 0.59; 95% CI, 0.45-0.77) and in the subgroup of RCTs comparing ICI use with chemotherapy (RR, 0.22; 95% CI, 0.13-0.39). In the subgroup of RCTs comparing ICI with placebo, the overall risk of NAE was significantly higher in the ICI group (RR, 1.57; 95% CI, 1.30-1.89). Peripheral neuropathy (RR, 0.30; 95% CI, 0.17-0.51) and dysgeusia (RR, 0.41; 95% CI, 0.27-0.63) were significantly lower in the ICI group. Headache was more common with the use of ICIs (RR, 1.32; 95% CI, 1.10-1.59). In the subgroup analysis of RCTs comparing ICI use with chemotherapy, peripheral neuropathy (RR, 0.09; 95% CI, 0.05-0.17), dysgeusia (RR, 0.42; 95% CI, 0.21-0.85), and paresthesia (RR, 0.29; 95% CI, 0.13-0.67) were significantly lower in the ICI group. RCTs comparing ICIs with placebo showed a higher risk of headache with ICI use (RR, 1.63; 95%, CI, 1.32-2.02).

**Conclusions and Relevance:**

Results of this meta-analysis suggest that the overall risk of NAEs, peripheral neuropathy, and dysgeusia is lower with the use of ICI. When compared with chemotherapy, the overall risk of NAE, peripheral neuropathy, paresthesia, and dysgeusia was lower with ICI use; however, when compared with placebo, the risk of NAEs is higher with the use of ICI.

## Key Points

**Question:**

What is the association of the use of checkpoint inhibitors with the risk of neurological adverse events?

**Findings:**

In this meta-analysis of 39 trials evaluating the use of checkpoint inhibitors to treat various malignant neoplasms, the risk of neurological adverse events was lower when compared with chemotherapy. However, the risk of neurologic adverse events was higher with checkpoint inhibitors compared with placebo.

**Meaning:**

These results suggest patients treated with checkpoint inhibitors are less likely to develop neurologic adverse events compared with other cancer medications, particularly cytotoxic chemotherapy.

## Teaser

This systematic review and meta-analysis compares neurologic adverse events among patients with cancer receiving immune checkpoint inhibitors compared with those receiving chemotherapy or placebo.

## Introduction

Immune checkpoint inhibitors (ICIs) have acquired a central role in the treatment of cancer within the last decade. Over 50 different tumor types have approval for the use of T-cell targeted immunomodulators blocking immune checkpoints like cytotoxic T-lymphocyte associated antigen-4 (CTLA-4), programmed death-1 (PD-1), or programmed death ligand 1 (PD-L1).^1^ However, because of their unique mechanism of action, ICIs present their own set of adverse events (AEs), called immune-related adverse events. Neurologic immune-related AEs are an emerging area of interest because of the complexity of the nervous system and the potential for long-term morbidity.^2,3^ Although the overall incidence of neurologic AE (NAE) is reported to be approximately 1%,^4^ NAEs constitute 11% of all the fatal events secondary to ICIs.^3^ In a systematic review of literature, Cuzzubbo et al^5^ reported that the overall incidence of NAEs of any grade was 3.8% with anti–CTLA-4, 6.1% for anti–PD-1/PD-L1, and 12% with the use of dual checkpoint inhibitors (a combination of anti–PD-1 and anti–CTLA-4 therapy).

The utility of checkpoint inhibitors is being increasingly explored in patients with brain tumors and brain metastases.^6,7^ Furthermore, multiple clinical trials are exploring the combination of ICIs with radiation therapy and oncolytic viruses to achieve better responses.^6,7^ Newer molecules inhibiting CTLA-4 or PD-1/PDL-1 are being studied in clinical trials treating multiple tumor types. In such a scenario, it becomes essential to understand the spectrum of NAEs and diagnose it at the onset to prevent long-term morbidity. However, ICI-related NAEs are reported only in systematic reviews and analysis of databases reporting adverse events. This study reports the first meta-analysis examining the NAEs reported in the randomized trials comparing ICIs with chemotherapy, targeted therapy, or placebo.

## Methods

### Data Sources and Study Selection

**Figure 1.**
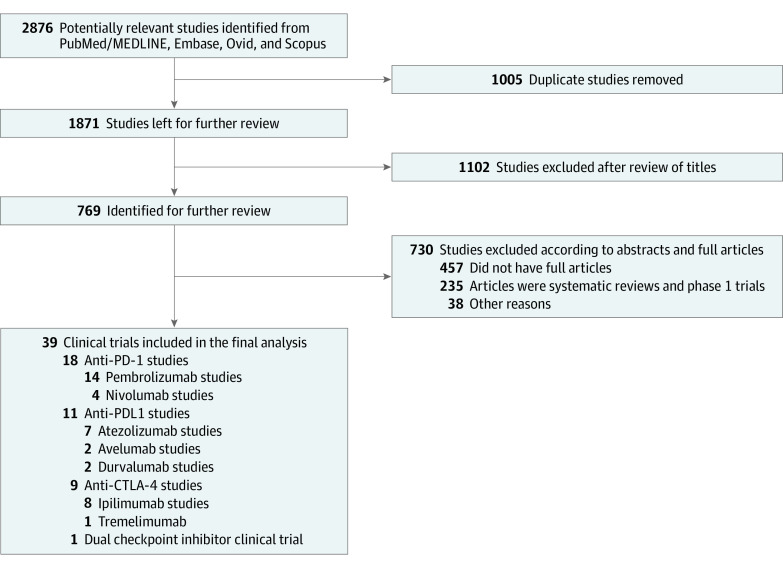
PRISMA Flowchart of Recruitment Strategy

In accordance with the Preferred Reported Items for Systematic Reviews and Meta-Analyses (PRISMA) reporting guideline (Figure 1),^8^ 2 authors (S.B.A. and P.L.) independently searched the bibliographic databases (Embase, Ovid, MEDLINE, and Scopus) and trial registries (ClinicalTrials.gov) from inception through March 1, 2020. Published trials fulfilling the PICO criteria (Participants, Intervention, Comparison, and Outcome) were included. Participants included adult patients with any cancer. The intervention included treatment with anti–PD-1/PD-L1 and/or anti-CTLA4 drugs. The comparison included drug regimens that used monotherapy or a combination of chemotherapy, targeted therapy, vaccines, or any medication used to treat that cancer. Trials using placebo or supportive care were also included in the comparison arm. Outcomes included NAEs of any grade or type. Only phase II and III randomized control trials (RCTs) comparing single or dual ICI with the standard of care or placebo were selected. Only those studies where full-text articles were available were screened for the final analysis. In the event of multiple publications from the same trial, only those with the largest sample size were included in our analysis. Any trials whose results were not published in a peer-reviewed journal were excluded. All databases were searched for publications containing anti–PD-1/PD-L1 or anti–CTLA-4 checkpoint inhibitors by using the search string: *ipilimumab* OR *tremelimumab* OR *nivolumab* OR *pembrolizumab* OR *MED10680* OR *AMP-224* OR *pidilizumab* OR *atezolimab* OR *MED14736* OR *avelumab* OR *BMS-936559* AND *durvalumab* OR *MEDI4736*. Two independent reviewers (P.F. and A.M.) examined all selected studies, and disagreements were resolved with mutual consensus or third-party review (M.Z.F.).

### Data Extraction and Quality Assessment

Four researchers (S.B.A., P.L., R.C.P., and S.U.B.) independently extracted the following data from the studies included in the analysis into Excel version 2020 (Microsoft Corp) using a standardized form: (1) study information, including the name of the first author, year of publication, category of the trial (phase II or III), interventions received, and the number of participants; (2) characteristics of participants, including median or mean age, gender, race and/or ethnicity, region, smoking status, and ECOG (Eastern Cooperative Oncology Group) performance status; and (3) NAEs of any grade and type, including altered mentation, cerebral edema, cerebrovascular accident, cranial nerve VII palsy, decreased appetite, dizziness, dysgeusia, encephalitis, fatigue, Guillain-Barré syndrome (GBS), headache, insomnia, intracranial hemorrhage, myasthenia gravis, myelitis, paraplegia, paresthesia, peripheral neuropathy, seizure, and trigeminal nerve disorder. To assess the quality of RCTs included in the analysis, 2 authors (S.K. and A.J.) used the Cochrane Collaborations tool (eTable 1 in the Supplement).^9^

We used the Common Terminology of Adverse Events (CTCAE) version 4.0 to define NAEs from systemic therapy as most protocols were written before November 2017 (when CTCAE version 5.0 was introduced). NAE includes the manifestation of any neurologic toxicity secondary to ICI use in the central nervous system, including the brain, brainstem, and spinal cord, extending to the peripheral nervous system, which includes the neuromuscular junction and muscle fibers. Severe toxic effects of the nervous systems included myasthenia gravis, GBS, transverse myelitis, encephalitis, and meningitis. According to CTCAE 4.0, the severity is represented by grades from 1 through 5, with unique clinical descriptions for each AE according to the severity.

### Statistical Analysis

Statistical heterogeneity was quantified using *I^2^* statistics with I values of 25%, 50%, and 75% deemed to represent low, moderate, and high heterogeneity, respectively. Otherwise, given the differences in the study populations, cancers being treated, the wide variety of chemotherapy and ICI agents being used in the individual trials, and the differences in the treatment effects, we expected significant heterogeneity between studies. A random-effects model of DerSimonian and Laird was used for all analyses. The estimates were reported as risk ratio (RR) with 95% CIs. To assess the stability of the pooled values, we performed sensitivity analyses. *P* > .05 was deemed the threshold for statistical significance in 2-sided tests. An assessment of publication bias was conducted based on a funnel plot (eFigure 1 in the Supplement). Visual inspection of the funnel plots suggested a low study bias. Our primary outcome was the assessment of NAE of all grades that included peripheral neuropathy, dizziness, headache, stroke, myasthenia gravis, GBS, myelitis, and encephalopathy based on previous reviews in the ICI group compared with the control group.^10,11,12^

## Results

### Eligible Studies and Study Characteristics

A total of 2876 full-text articles retrieved from the initial database search were analyzed according to PRISMA guidelines (Figure 1). After the final analysis, 39 trials^13,14,15,16,17,18,19,20,21,22,23,24,25,26,27,28,29,30,31,32,33,34,35,36,37,38,39,40,41,42,43,44,45,46,47,48,49,50,51,52,53,54,55,56,57,58,59,60,61,62,63,64,65^ met our inclusion criteria, which comprised 13 110 patients in the control arm and 10 595 patients in the ICI arm (Table 1). Analysis involved 16 RCTs evaluating ICI use treating patients with non–small cell lung cancer that included 9074 patients, subclassification of which showed 4964 in the ICI arm and 4110 in the control arm. By cancer type, 5 RCTs evaluating patients with melanoma, 4 RCTs evaluating patients with renal cell carcinoma, 3 RCTs evaluating multiple myeloma, 2 RCTs each evaluating small cell lung cancer and head and neck cancer, and 1 RCT each evaluating patients with colorectal, gastric, bladder, gastroesophageal junction, urothelial cancer, prostate, and mesothelioma were included in the final analysis.

**Table 1.** Characteristics of Included Clinical Trials

Source	Phase/study design	Tumor type	No. of patients	Dose of checkpoint inhibitor	Kind of CPI	Previous treatment	Treatment
Chih-Hsin Yang et al,^31^ 2019 (CAURAL Study group)	Phase III (1:1)	Advanced NSCLC	29	Durvalumab 10 mg/kg	PD-L1	Yes (EGFR-TKI)	Durvalumab + osimertinib vs osimertinib
Cohen et al,^32^ 2019 (KEYNOTE-040)	Phase III (1:1)	Recurrent/relapsed HNSCC	495	Pembrolizumab 200 mg	PD-1	Yes (Platinum therapy)	Pembrolizumab vs SOC
Eng et al,^33^ 2019 (IMblaze370)	Phase III (2:1:1)	Metastatic colorectal cancer	363	Atezolizumab 840 mg	PD-L1	Yes (≥2 previous therapies)	Atezolizumab + cobimetinib vs atezolizumab monotherapy vs regorafenib monotherapy
Mateos et al,^34^ 2019 (KEYNOTE-183)	Phase III (1:1)	Multiple myeloma	249	Pembrolizumab 200 mg	PD-1	Yes (≥2 previous therapies)	Pembrolizumab + pomalidomide + dexamethasone vs pomalidomide + dexamethasone
Mok et al,^35^ 2019 (KEYNOTE-042)	Phase III (1:1)	Locally advanced or metastatic NSCLC	1274	Pembrolizumab 200 mg	PD-1	No	Pembrolizumab vs platinum-based chemotherapy
Rini et al,^36^ 2019 (IMmotion151)	Phase III (1:1)	Metastatic RCC	915	Atezolizumab 1200 mg	PD-L1	No	Atezolizumab + bevacizumab vs sunitinib
Rini et al,^37^ 2019 (KEYNOTE-426)	Phase III (1:1)	Locally advanced or metastatic RCC	861	Pembrolizumab 200 mg	PD-1	No	Pembrolizumab + axitinib vs sunitinib
Usmani et al,^15^ 2019 (KEYNOTE-185)	Phase III (1:1)	Multiple myeloma	310	Pembrolizumab 200 mg	PD-1	No	Pembrolizumab + lenalidomide + dexamethasone vs lenalidomide + dexamethasone
West et al,^38^ 2019 (IMpower130)	Phase III (1:1)	Stage IV, nonsquamous, NSCLC	723	Atezolizumab 1200 mg	PD-L1	No	Atezolizumab + nab-paclitaxel + carboplatin vs nab-paclitaxel + carboplatin
Bang et al,^39^ 2019 (JAVELIN Gastric 300)	Phase III (1:1)	Advanced gastric cancer/GEJ	371	Avelumab 10 mg/kg	PD-L1	Yes (2 lines of treatment)	Avelumab vs paclitaxel or irinotecan or best supportive care
Barlesi et al,^40^ 2019 (JAVELIN Lung 200)	Phase III (1:1)	Stage IIIB/IV or recurrent NSCLC	792	Avelumab 10 mg/kg	PD-L1	Yes (Platinum-based doublet)	Avelumab vs docetaxel
Eggermont et al,^41^ 2019 (KEYNOTE-054)	Phase III (1:1)	Stage III melanoma	1019	Pembrolizumab 200 mg	PD-1	No	Pembrolizumab vs placebo
Gandhi et al,^42^ 2019 (KEYNOTE-189)	Phase III (2:1)	Metastatic non-squamous NSCLC	616	Pembrolizumab 200 mg	PD-1	No	Pembrolizumab + pemetrexed + platinum vs first-line chemotherapy (physician’s choice)
Horn et al,^43^ 2019 (IMpower133)	Phase III (1:1)	Extensive stage small-cell lung cancer	403	Atezolizumab 1200 mg	PD-L1	No	Atezolizumab + carboplatin + etoposide vs carboplatin + etoposide + placebo
Motzer et al,^14^ 2018 (CHECKMATE-214)	Phase III (1:1)	Advanced or metastatic RCC	1390	Nivolumab 3 mg/kg + Ipilimumab 1 mg/kg	Dual	No	Ipilimumab + nivolumab vs sunitinib
Paz-Ares et al,^44^ 2018 (KEYNOTE-407)	Phase III (1:1)	Metastatic squamous NSCLC	559	Pembrolizumab 200 mg (35 cycles)	PD-1	No	Pembrolizumab + carboplatin + paclitaxel or nab-paclitaxel vs carboplatin + paclitaxel or nab-paclitaxel
Powles et al,^45^ 2018 (IMvigor211)	Phase III (1:1)	Locally advanced or metastatic urothelial bladder cancer	931	Atezolizumab 1200 mg	PD-L1	Yes (Platinum-based regimen)	Atezolizumab vs physician choice chemo (vinflunine, paclitaxel, or docetaxel)
Shitara et al,^46^ 2018 (KEYNOTE 061)	Phase III (1:1)	Advanced gastric cancer/GEJ	395	Pembrolizumab 200 mg	PD-1	Yes (Platinum and 5-FU)	Pembrolizumab vs paclitaxel
Antonia et al,^47^ 2017 (PACIFIC)	Phase III (2:1)	Stage III NSCLC	713	Durvalumab 10 mg/kg	PD-L1	No	Durvalumab vs placebo
Bang et al,^48^ 2017	Phase II (1:1)	Advanced gastric cancer/GEJ	143	Ipilimumab 10 mg/kg	CTLA-4	Yes (≥1 line of chemotherapy)	Ipilimumab vs supportive care (continue 5-FU)
Bellmunt et al,^49^ 2017 (KEYNOTE-045)	Phase III (1:1)	Recurrent or metastatic urothelial carcinoma	542	Pembrolizumab 200 mg	PD-1	Yes	Pembrolizumab vs docetaxel/paclitaxel or vinflunine
Carbone et al,^50^ 2017 CHECKMATE 026	Phase III	Stage IV or recurrent NSCLC	1325	Nivolumab 3 mg/kg	PD-1	No	Nivolumab vs gemcitabine/paclitaxel/pemetrexed
Govindan et al,^51^ 2017	Phase III (1:1)	Stage IV/ recurrent squamous NSCLC	749	Ipilimumab 10 mg/kg	CTLA-4	No	Ipilimumab + paclitaxel/carboplatin vs placebo + paclitaxel/carboplatin
Hamid et al,^52^ 2017 (KEYNOTE 002)	Phase II (1:1:1)	Advanced melanoma	540	Pembrolizumab 2 mg/kg and 10 mg/kg	PD-1	Yes (Ipilimumab or BRAF/MEK inhibitors or both)	Pembrolizumab 2 mg/kg vs pembrolizumab 10 mg/kg vs carboplatin/paclitaxel/dacarbazine/temozolomide
Maio et al,^53^ 2017 (DETERMINE)	Phase IIb (2:1)	Relapsed mesothelioma	571	Tremelimumab 10 mg/kg	CTLA-4	Yes (1 or 2 lines of therapy)	Tremelimumab vs placebo
Rittmeyer et al,^54^ 2017 (OAK study group)	Phase III (1:1)	Stage IIIB/ IV or recurrent NSCLC	1225	Atezolizumab 1200 mg	PD-L1	Yes (1 or 2 platinum-based regimens)	Atezolizumab vs docetaxel
Fehrenbacher et al,^55^ 2016 (POPLAR)	Phase II (1:1)	Stage IIIB/IV or recurrent NSCLC	287	Atezolizumab 1200 mg	PD-L1	Yes (Platinum-based regimen)	Atezolizumab vs docetaxel
Ferris et al,^56^ 2016 (CHECKMATE-141)	Phase III (2:1)	Recurrent or stage III/IV HNSCC	506	Nivolumab 3 mg/kg	PD-1	Yes (Platinum-based regimen)	Nivolumab vs cetuximab or methotrexate or docetaxel
Herbst et al,^57^ 2016 (KEYNOTE-010)	Phase II/III (1:1:1)	Advanced NSCLC	1034	Pembrolizumab 2 mg/kg and 10 mg/kg	PD-1	Yes (platinum-containing doublet)	Pembrolizumab 2 mg/kg vs pembrolizumab 10 mg/kg vs docetaxel
Langer et al,^58^ 2016 (KEYNOTE-021)	Phase II, (1:1)	Stage IIIB/IV non-squamous NSCLC	123 (1:1)	Pembrolizumab 200 mg	PD-1	No	Pembrolizumab with carboplatin + pemetrexed vs carboplatin + pemetrexed
Reck et al,^59^ 2016 (KEYNOTE-024)	Phase III, (1:1)	Advanced NSCLC	305 (1:1)	Pembrolizumab 200 mg	PD-1	No	Pembrolizumab vs platinum-based chemotherapy
Reck et al,^60^ 2016	Phase III, (1:1)	Small cell lung cancer	1132 (1:1)	Ipilimumab 10 mg/kg	CTLA-4	No	Ipilimumab + platinum + etoposide vs placebo + platinum + etoposide
Borghaei et al,^61^ 2015 (CHECKMATE-057)	Phase III (1:1)	NSCLC	582 (1:1)	Nivolumab 3 mg/kg	PD-1	Yes (Platinum-based doublet therapy)	Nivolumab vs docetaxel
Eggermont et al,^62^ 2015 (EORTC 18071)	Phase III (1:1)	High-risk stage III melanoma	951 (1:1)	Ipilimumab 10 mg/kg	CTLA-4	No	Ipilimumab vs placebo
Motzer et al,^13^ 2015 (CHECKMATE-025)	Phase III (1:1)	Advanced RCC	821 (1:1)	Nivolumab 3 mg/kg	PD-1	1-2 lines of antiangiogenic therapy	Nivolumab vs everolimus
Kwon et al,^63^ 2014 (CA184-043)	Phase III (1:1)	Stage IV, castration resistant prostate cancer	799 (1:1)	Ipilimumab 10 mg/kg	CTLA-4	Yes (Docetaxel)	Ipilimumab vs placebo
Reck et al,^64^ 2013	Phase II (1:1:1)	Extensive small cell lung cancer	130 (1:1:1)	Ipilimumab 10 mg/kg	CTLA-4	No	Ipilimumab + carboplatin/paclitaxel (concurrent) vs ipilimumab + carboplatin/paclitaxel (phased) vs placebo + carboplatin/paclitaxel
Robert et al,^65^ 2011	Phase III (1:1)	Stage IV melanoma	502 (1:1)	Ipilimumab 10 mg/kg	CTLA-4	No	Ipilimumab + dacarbazine vs placebo + dacarbazine
Hodi et al,^16^ 2010	Phase III (3:1:1)	Stage IV/unresectable stage III melanoma	676 (3:1:1)	Ipilimumab 3 mg/kg	CTLA-4	Yes	Ipilimumab + gp100 vs ipilimumab monotherapy vs gp100 monotherapy

Abbreviations: CPI, checkpoint inhibitor; CTLA-4, cytotoxic T-lymphocyte antigen-4; GEJ, gastroesophageal junction; gp100, glycoprotein-100; HNSCC, head and neck squamous cell carcinoma; NSCLC, non–small cell lung cancer; PD-1, programmed cell death protein-1; PD-L1, programmed cell death ligand protein-1; RCC, renal cell cancer.

To explore the differences in NAEs between patients receiving ICIs and other treatments or placebo, we performed a subgroup analysis of the RCTs that only had ICIs in 1 arm and non-ICI drugs or placebo in the other arm (for example, a trial comparing the combination of chemotherapy and ICIs with chemotherapy alone or ICIs alone was excluded from subgroup analysis). One trial each comparing ICI use with nonchemotherapy drugs, namely, everolimus (CHECKMATE-025^13^), sunitinib (KEYNOTE-426^37^), lenalidomide (KEYNOTE-185^15^), sunitinib (IMmotion-151^36^), and glycoprotein-100 (Hodi et al^16^) were reporting NAEs. Due to the differences in the mechanism of action of these drugs, we did not analyze them separately as a subgroup. This study also included subgroup analyses of RCTs exploring the efficacy of ICI with chemotherapy (15 trials) and ICI with placebo (5 trials).

### Overview and Demographics

NAEs were reported in 1989 patients (15.2%) in the ICI group and in 2110 patients (19.9%) in the comparative arm (Table 2). In the ICI group, 1400 NAEs (70.4%) were reported with the anti–PD-1/PDL-1 agents compared with 504 events (25.3%) with the anti–CTLA-4 group. The trial reported by Motzer et al^14^ evaluated a combination of anti–PD-1/PDL-1 and anti–CTLA-4 agents, reporting cumulative neurotoxicity in 314 patients. Demographic information was only reported in the intention-to-treat (ITT) population (23 733 patients). Among the ITT population, 16 135 [68.0%] were men, and 7866 [33.1%] were categorized as White individuals. The majority of patients were diagnosed with non–small cell lung cancer in both the control group (4060 [37.6%]) and the ICI group (5116 [39.5%]). (The demographics of the patients in the ITT population are listed in eTable 2 in the Supplement.)

**Table 2.** Summary of Neurological Adverse Events Reported in the Respective Trials

Adverse event	Overall neurotoxicity			ICI vs chemotherapy			ICI vs placebo		
	No. of trials	ICI, No. (%)	Comparator arm, No. (%)	No. of trials	ICI, No. (%)	Chemotherapy, No. (%)	No. of Trials	ICI, No. (%)	Placebo, No. (%)
Overall events	39	1989 (15.0)	2110 (19.9)	15	317 (6.0)	757 (17.4)	5	389 (17.5)	223 (12.4)
Paresthesia	10	112 (3.0)	115 (4.4)	5	27 (1.3)	63 (4.4)	2	26 (3.4)	12 (2.1)
Peripheral neuropathy	23	302 (3.9)	622 (10.2)	13	72 (1.4)	447 (10.8)	2	6 (0.8)	9 (1.5)
Headache	5	736 (11.7)	406 (8.9)	5	68 (3.5)	32 (2.2)	3	211 (16.97)	124 (11.7)
Dysgeusia	16	299 (4.9)	704 (14.4)	8	59 (1.9)	162 (6.5)	0	NA	NA
Dizziness	11	241 (6.6)	112 (4.7)	5	51 (2.8)	29 (2.5)	2	41 (5.30)	21 (3.6)
Insomnia	7	161 (6.2)	81 (4.4)	4	63 (4.0)	30 (2.8)	1	31 (7.89)	0
Altered mental status	7	76 (3.1)	37 (2.1)	3	2 (0.2)	1 (0.1)	2	51 (6.60)	34 (5.8)
Rare neurological adverse events (≤1% incidence)									
Encephalopathy	7	14 (0.6)	2 (0.1)	2	2 (0.3)	0	2	1 (0.26)	1 (0.4)
CVA	5	17 (0.9)	11 (0.9)	1	1 (0.4)	0	2	6 (0.69)	6 (1.0)
Seizures	4	14 (0.9)	4 (0.4)	1	1 (0.4)	0	2	6 (0.78)	3 (0.5)
GBS	5	5 (0.3)	0	1	1 (0.4)	0	2	2 (0.2)	0
Cranial nerve VII paresis	1	2 (0.5)	0	0	NA	NA	0	NA	NA
Intracranial hemorrhage	1	2 (0.5)	4 (1.0)	0	NA	NA	1	2 (0.5)	4 (1.0)
Cerebral edema	2	7 (0.8)	3 (0.7)	0	NA	NA	1	1 (0.3)	0
Myelitis	1	0	1 (0.3)	0	NA	NA	1	0	1 (0.3)
Myasthenia gravis	1	1 (0.7)	0	0	NA	NA	0	NA	NA
Trigeminal neuralgia	2	1 (0.2)	1 (0.2)	0	NA	NA	1	0	1 (0.3)
Paraplegia	2	4 (0.5)	7 (1.2)	0	NA	NA	2	4 (0.5)	7 (1.2)

Abbreviations: CVA, cerebrovascular accidents; GBS, Guillain-Barré Syndrome; ICI, immune checkpoint inhibitors; NA, not applicable.

### Meta-analysis of the Outcomes

#### ICI vs Control Group

**Figure 2.**
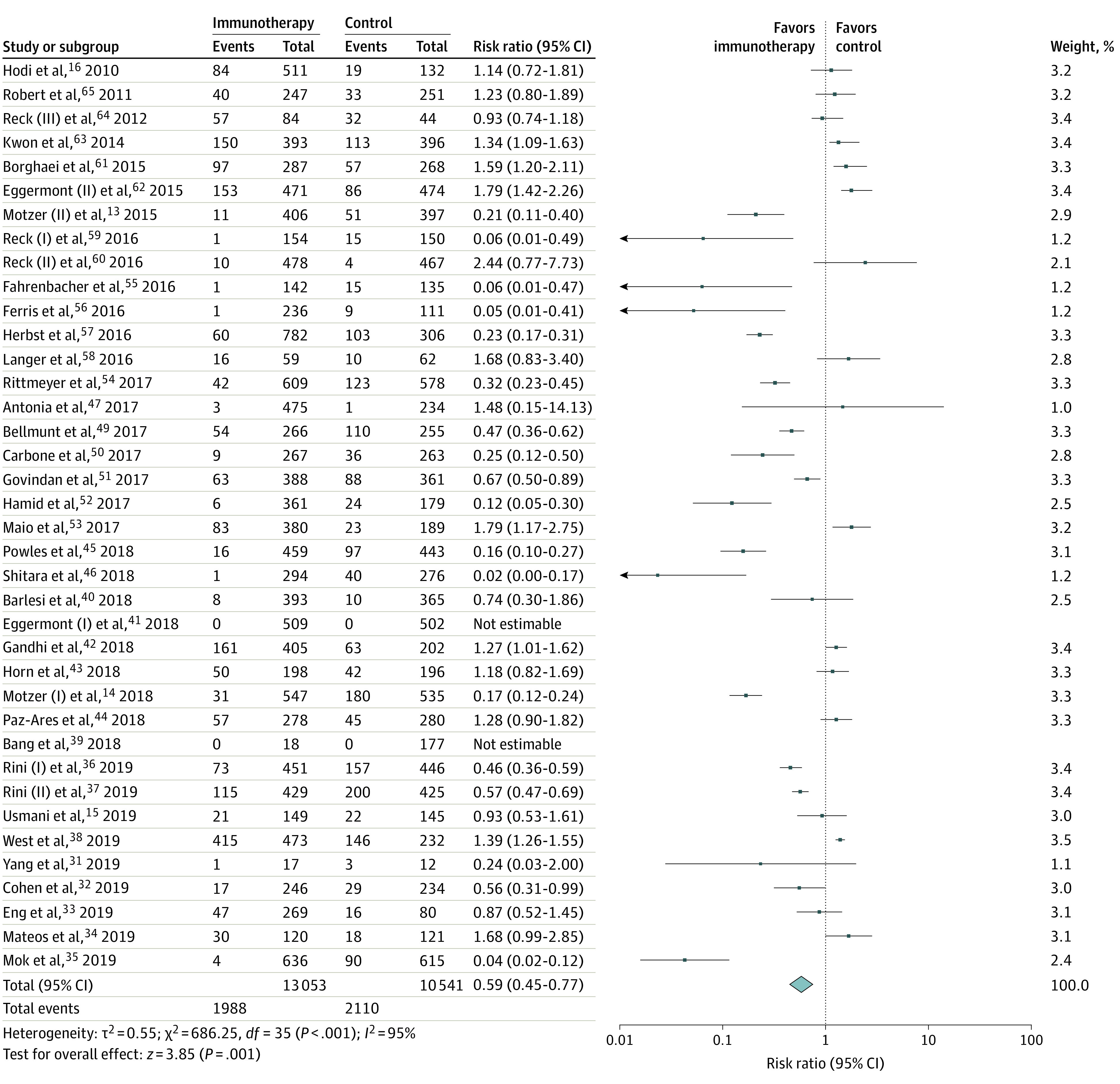
Neurotoxicity Analysis of Checkpoint Inhibitors vs Control

Overall, we compared the risk of NAEs with treatment using ICIs compared with the control arm (comprising trials using drug regimens including chemotherapy, targeted therapy, vaccines, or combination therapies) or placebo. All-grade NAEs were significantly lower with ICIs compared with the control arm (15.0% vs 19.9%; RR, 0.59; 95% CI, 0.45-0.77; *I^2^* = 95%, *P* < .001) among all recruited studies (Figure 2). Twenty-three trials reported peripheral neuropathy, which was significantly lower in patients in the ICI group compared with those in the control group (4.2% vs 10.5%; RR, 0.30; 95% CI, 0.17-0.51; *I^2^* = 91%; *P* < .001) (eFigure 2 in the Supplement). More patients in the ICI group reported headache than the control group (11.6% vs 8.8%; RR, 1.32; 95% CI, 1.10-1.59; *I^2^* = 51%; *P* = .008). Fewer patients in the ICI arm reported dysgeusia compared with those in the control group (4.9% vs 14.4%; RR, 0.41; 95% CI, 0.27-0.63; *I^2^* = 83%; *P* < .001).

Eleven RCTs reported dizziness with 6.6% of patients in the ICI arm and 4.7% in the control arm (RR, 1.16; 95% CI, 0.75-1.79; *I^2^* = 64%; *P* = .50). Seven trials reported altered mental status with 3.9% of patients in the ICI group and 2.1% in the control (RR, 1.30; 95% CI, 0.85-1.97; *I^2^* = 2%; *P* = .22). Paresthesia was reported in 10 trials with 3.0% of patients in the ICI group and 4.4% in the control arm (RR, 0.61; 95% CI, 0.31-1.23; *I^2^* = 79%; *P* = .17). Seven trials reported insomnia with 6.2% of patients in the ICI group and 4.4% in the control arm (RR, 1.40; 95% CI, 0.66-2.99; *I^2^* = 79%; *P* = .38) (eFigure 2 in the Supplement). Rare events reported in the trials are listed in Table 2. These data points were not analyzed further because of the rarity of the events.

We analyzed the overall risk of NAE after removing the incidence of peripheral neuropathy from both the ICI and the control arm. The overall risk of NAE was lower in the ICI arm compared with the control arm (12.8% vs 14.0%; RR, 0.74; 95% CI, 0.56-0.97; *I^2^* = 93%; *P* = .03] (eFigure 5 in the Supplement)

### Subgroup Analysis

#### ICI vs Chemotherapy

**Figure 3.**
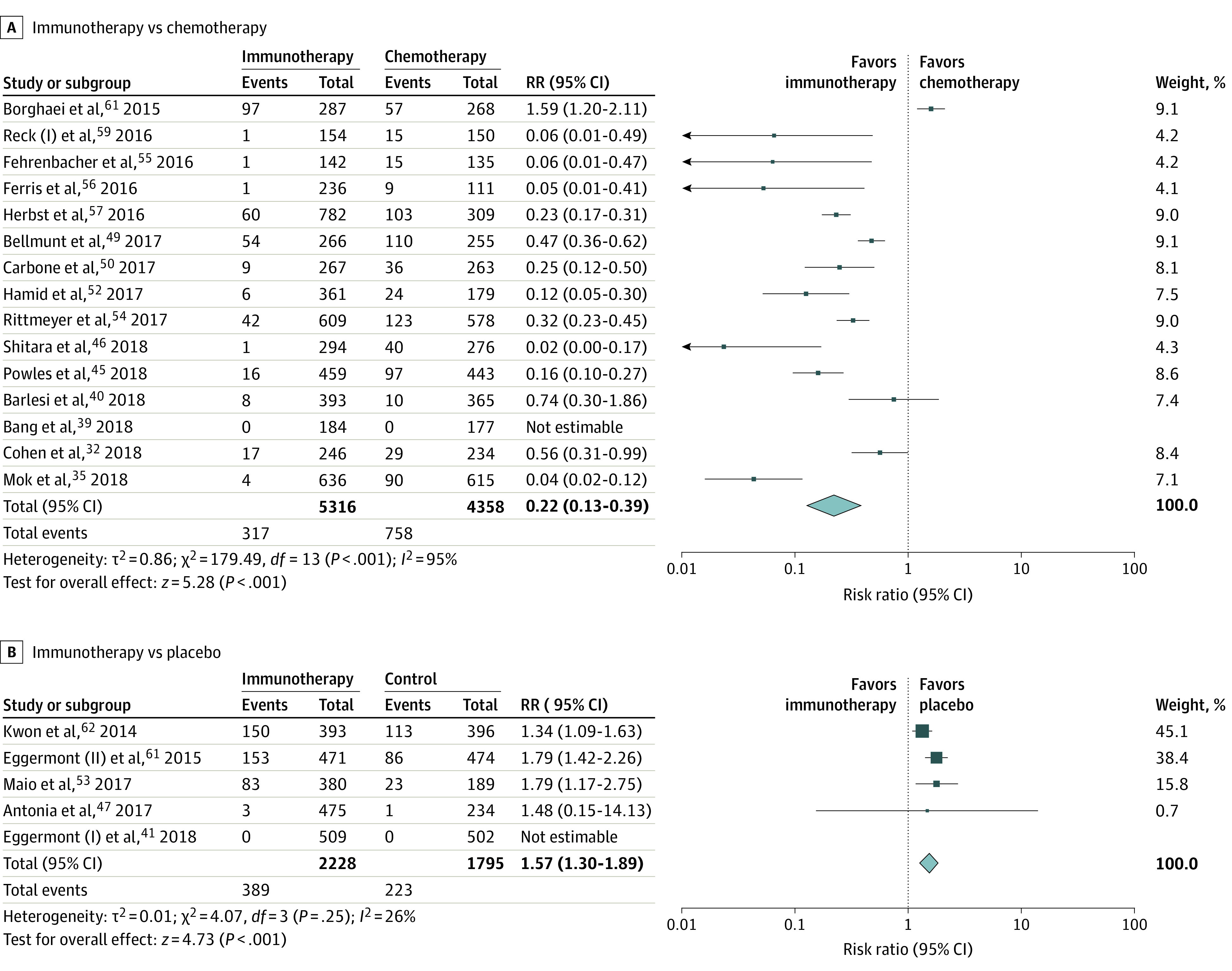
Overall Neurotoxicity in the Subgroup Analysis (A) Immunotherapy vs Chemotherapy and (B) Immunotherapy vs Placebo

Fifteen trials comparing ICI use with chemotherapy were analyzed separately. NAEs were reported in 317 patients (6.0%) in the ICI arm and 757 patients (17.4%) in the chemotherapy arm. The overall risk of NAE was significantly lower in the ICI group compared with the chemotherapy arm (RR, 0.22; 95% CI, 0.13-0.39; *I^2^* = 93%; *P* < .001) (Figure 3A). Twelve trials reported a significantly lower risk of peripheral neuropathy in the ICI arm vs chemotherapy arm (1.4% vs 10.8%; RR, 0.09; 95% CI, 0.05-0.17; *I^2^* = 74%; *P* < .001) (eFigure 3 in the Supplement). Ten trials reported a significantly lower risk of dysgeusia in the ICI arm vs chemotherapy arm (1.9% vs 6.5%; RR, 0.42; 95% CI, 0.21-0.85; *I^2^* = 68%; *P* = .02). Five trials reported a significantly lower risk of paresthesia in the ICI arm vs chemotherapy arm (1.3% vs 4.4%; RR, 0.29; 95% CI, 0.13-0.67; *I^2^* = 58%; *P* = .003) (eFigure 3 in the Supplement).

Five trials reported headache with 3.5% of patients in the ICI group and 2.2% in the chemotherapy arm (RR, 1.66; 95 CI, 0.61-4.46; *I^2^* = 75%; *P* = .32). Four trials reported insomnia with 4.0% of patients in the ICI group and 2.8% in the chemotherapy arm (RR, 1.39; 95% CI, 0.32-5.97; *I^2^* = 81%; *P* = .66). Five trials reported dizziness with 2.8% of patients in the ICI group and 2.5% in the chemotherapy arm (RR, 0.98; 95% CI, 0.24-3.91; *I^2^* = 75%; *P* = .97) (eFigure 3 in the Supplement). Rare events with an incidence of less than 1% were not analyzed (Table 2). We analyzed the overall risk of NAEs after removing the incidence of peripheral neuropathy from both the ICI and the chemotherapy arm. The overall risk of NAEs was lower with the ICI arm compared with the chemotherapy arm (4.8% vs 7.9%; RR, 0.47; 95% CI, 0.56-0.97; *I^2^* = 90%; *P* < .001) (eFigure 5 in the Supplement).

#### ICI vs Placebo

Five trials comparing ICI with placebo were analyzed separately. NAEs were reported in 389 patients (17.5%) in the ICI arm and 223 patients (12.4%) in the placebo arm. The overall risk of NAE was higher in the ICI group compared with the placebo group (RR, 1.57; 95% CI, 1.30-1.89; *I^2^* = 26%; *P* = .25) (Figure 3B). Three trials reported a significantly higher risk of headache with ICI (RR, 1.63; 95% CI, 1.32-2.02; *I^2^* = 4%; *P* = .35) (eFigure 4 in the Supplement). These results are considered statistically significant due to a low number of studies in the metanalysis, where confidence interval is considered a superior measure of determining significance.^17^ Rare events (like myasthenia gravis, and GBS) and events reported by less than 3 trials were not analyzed (Table 2).

## Discussion

To the best of our knowledge, this is the first meta-analysis of NAEs reported in RCTs using ICIs. The overall risk of NAEs was significantly lower in the ICI group. However, the heterogeneity in the comparator arm limits the interpretation of this analysis as several RCTs involved either chemotherapy in both arms or had an immunomodulator, tyrosine kinase inhibitor, or placebo in the comparator arm. Also, most of these trials included patients with relapsed refractory neoplasms who have had previous chemotherapy exposure, which could have contributed to the increased incidence of NAEs. The subgroup analysis of ICIs vs chemotherapy included trials in which patients either received ICIs or chemotherapy. This analysis was done to nullify the confounding effect of having chemotherapy in both arms and to clearly understand the association of NAEs with ICI use. Fifteen trials included in this subgroup showed a significantly lower risk of NAEs in the ICI subgroup. Lastly, the subgroup analysis comparing ICIs with placebo was done to assess the association of ICIs with NAEs in trials where patients in the comparator arm did not receive any treatment or received placebo. This meta-analysis showed a significantly higher risk of NAEs with ICIs than those who received a placebo. These results indicated that although ICI use is associated with an increased risk of NAEs, the risk is much lower when compared with chemotherapy.

The neurologic toxicity in the peripheral nervous system associated with ICI use includes mild to moderate peripheral neuropathy or more catastrophic events like GBS, myositis, and myasthenia gravis.^12,18^ Acute or chronic peripheral neuropathy occurs in up to 3% of all patients treated with ICIs.^4,5,11,19^ In our analysis, the overall risk of peripheral neuropathy was significantly lower in the ICI arm than in the control arm. In the subgroup analysis of RCTs comparing ICIs with chemotherapy, peripheral neuropathy and paresthesia were significantly higher in the chemotherapy arm. A recent pharmacovigilance study reported a higher risk of peripheral neuropathy with the use of ICI. However, the authors acknowledged that they had included GBS in the definition of peripheral neuropathy.^20^ In RCTs, peripheral neuropathy and paresthesia are defined by symptoms that arise from damage to the peripheral motor or sensory neurons according to CTCAE criteria. Five trials reported 1 patient each with GBS separate from those who developed peripheral neuropathy. Also, most of the trials in the comparison arms included patients who either received taxanes and platinum during the trial or had received them in previous lines of treatment. The incidence of chemotherapy-induced peripheral neuropathy is estimated to be nearly 19% to 85% depending on the study and highest among patients exposed to taxanes (11% to 87%) and platinum (70% to 100%).^21,22,23^ This explains the lower risk of peripheral neuropathy in both the overall analysis and the subgroup analysis of ICIs vs chemotherapy. We also performed the meta-analysis of all trials and those comparing ICIs with chemotherapy after removing the incidence of peripheral neuropathy (eFigure 5 in the Supplement). The risk of NAEs remains lower with ICIs, which indicates that peripheral neuropathy alone does not skew our results.

Dysgeusia occurs due to alteration in the taste receptor cells and taste bud cells, which is more common with chemotherapy.^24^ We noted a significantly lower risk of dysgeusia with ICI in the overall analysis and in the subgroup of ICIs vs chemotherapy. Retrospective studies have reported dysgeusia in up to 1.6% of patients with ICIs.^25^ On the other hand, up to 67% of patients receiving chemotherapy develop dysgeusia of any grade, and 38% of patients develop moderate to severe dysgeusia.^26,27^ Our results demonstrate that although dysgeusia can occur with ICIs, the risk is lower compared with chemotherapy. Headache is one of the common NAEs from the use of ICIs. It is either associated with the endocrine AEs seen with ICIs or as a separate neurological event.^28^ The subgroup analysis of ICIs vs placebo showed a significantly increased risk of headache compared with the placebo group, strengthening the association with ICIs as the causative factor. The use of chemotherapy also increases the risk of having a headache. Therefore the results were not statistically significant in the subgroup of ICIs vs chemotherapy despite a higher incidence of headache with the use of ICIs.

RCTs do not capture rarer adverse events reliably.^29^ Myasthenic syndrome is a rare NAE of the peripheral nervous system and is often associated with the highest mortality among all NAEs.^3,20^ Although our analysis reports only 1 patient in the ICI arm to develop myasthenia gravis, studies from various databases and retrospective reviews indicate the frequency as high as 0.1 to 0.47%.^5,11,12,20^ GBS-like syndromes occur in 0.1% to 0.2% of all patients treated with ICI.^12,20^ In our analysis, only 5 patients in the ICI arm developed GBS-like syndrome. The use of ICIs among the general population is quite different from a controlled environment of an RCT; hence rarer AEs are often better elucidated in database studies.^30^

### Limitations and Strength

The study has a few limitations. First, in analyzing the cumulative risk of NAEs, several trials are included where chemotherapy was used in both arms, which could lead to an overestimation of risk in the ICI arm. The subgroup analyses of ICIs vs chemotherapy and ICIs vs placebo was done to overcome this limitation. Second, the considerable differences among trials in patient characteristics, studied intervention, cointerventions or background therapy, or outcome assessment likely led to considerable heterogeneity in this meta-analysis. However, reassuringly, in most of the analyses the differences between studies were in the magnitude and not the direction of effects. We ensured strict adherence to the inclusion and exclusion criteria. The third limitation of our study comes from the exclusion of data from unpublished studies, which could introduce what could be described as a “file-drawer problem.” It is hard to check the accuracy of unpublished empirical data, especially when it does not undergo the rigors of the peer review process. We have presented funnel plots (eFigure 1 in the Supplement) to help the reader understand the publication bias of the included studies. Lastly, as mentioned above, RCTs do not capture rare NAEs adequately. Hence, we cannot reliably analyze these events in this meta-analysis.

## Conclusions

This meta-analysis found that the overall risk of NAEs was lower with ICIs than in control groups (containing chemotherapy, targeted therapy, placebo, etc). Subgroup analysis showed that overall, NAEs (including peripheral neuropathy, headache, and dysgeusia) were less common with ICIs than chemotherapy. However, compared with placebo, ICIs were associated with a higher risk of NAEs. Further research is needed to understand the full spectrum of NAEs associated with the use of ICIs, especially the rarer NAEs that are not commonly registered in RCTs.

## Notes

Supplement. **eFigure 1.** Funnel Plots for Included Trials by Subgroup **eFigure 2.** Overall Analysis of Incidence of Adverse Events Between Checkpoint Inhibitor and Control Arm **eFigure 3.** Subgroup Analysis of Adverse Events Between Checkpoint Inhibitors and Chemotherapy **eFigure 4.** Subgroup Analysis of Headache Between Checkpoint Inhibitors and Placebo **eFigure 5.** Overall Risk of Neurologic Adverse Events Excluding Incidence of Specific Event Between Checkpoint Inhibitors and Control Arm **eTable 1.** Quality Assessment of All Trials Using the Cochrane Collaborations Tool **eTable 2.** Pooled Characteristics of the Intention-to-Treat Population
