# Supplementary material for: Association of Immune Checkpoint Inhibitors With Neurologic Adverse Events: A Systematic Review and Meta-analysis
Source: JAMA Netw Open. 2022 Apr 19;5(4):e227722. doi: 10.1001/jamanetworkopen.2022.7722 (PMC9020216; doi:10.1001/jamanetworkopen.2022.7722)
Supplement: Supplement. — eFigure 1. Funnel Plots for Included Trials by Subgroup eFigure 2. Overall Analysis of Incidence of Adverse Events Between Checkpoint Inhibitor and Control Arm eFigure 3. Subgroup Analysis of Adverse Events Between Checkpoint Inhibitors and Chemotherapy eFigure 4. Subgroup Analysis of Headache Between Checkpoint Inhibitors and Placebo eFigure 5. Overall Risk of Neurologic Adverse Events Excluding Incidence of Specific Event Between Checkpoint Inhibitors and Control Arm eTable 1. Quality Assessment of All Trials Using the Cochrane Collaborations Tool eTable 2. Pooled Characteristics of the Intention-to-Treat Population [file jamanetwopen-e227722-s001.pdf]

Supplemental Online Content

Farooq MZ, Aqeel SB, Lingamaneni P, et al. Association of immune checkpoint inhibitors with neurologic adverse events: a systematic review and meta-analysis. *JAMA Netw Open.* 2022;5(4):e227722. doi:10.1001/jamanetworkopen.2022.7722

eFigure 1. Funnel Plots for Included Trials by Subgroup

eFigure 2. Overall Analysis of Incidence of Adverse Events Between Checkpoint Inhibitor and Control Arm

eFigure 3. Subgroup Analysis of Adverse Events Between Checkpoint Inhibitors and Chemotherapy

eFigure 4. Subgroup Analysis of Headache Between Checkpoint Inhibitors and Placebo

eFigure 5. Overall Risk of Neurologic Adverse Events Excluding Incidence of Specific Event Between Checkpoint Inhibitors and Control Arm

eTable 1. Quality Assessment of All Trials Using the Cochrane Collaborations Tool

eTable 2. Pooled Characteristics of the Intention-to-Treat Population

This supplemental material has been provided by the authors to give readers additional information about their work.

© 2022 Farooq MZ et al. *JAMA Network Open.*

eFigure 1. Funnel Plots for Included Trials by Subgroup

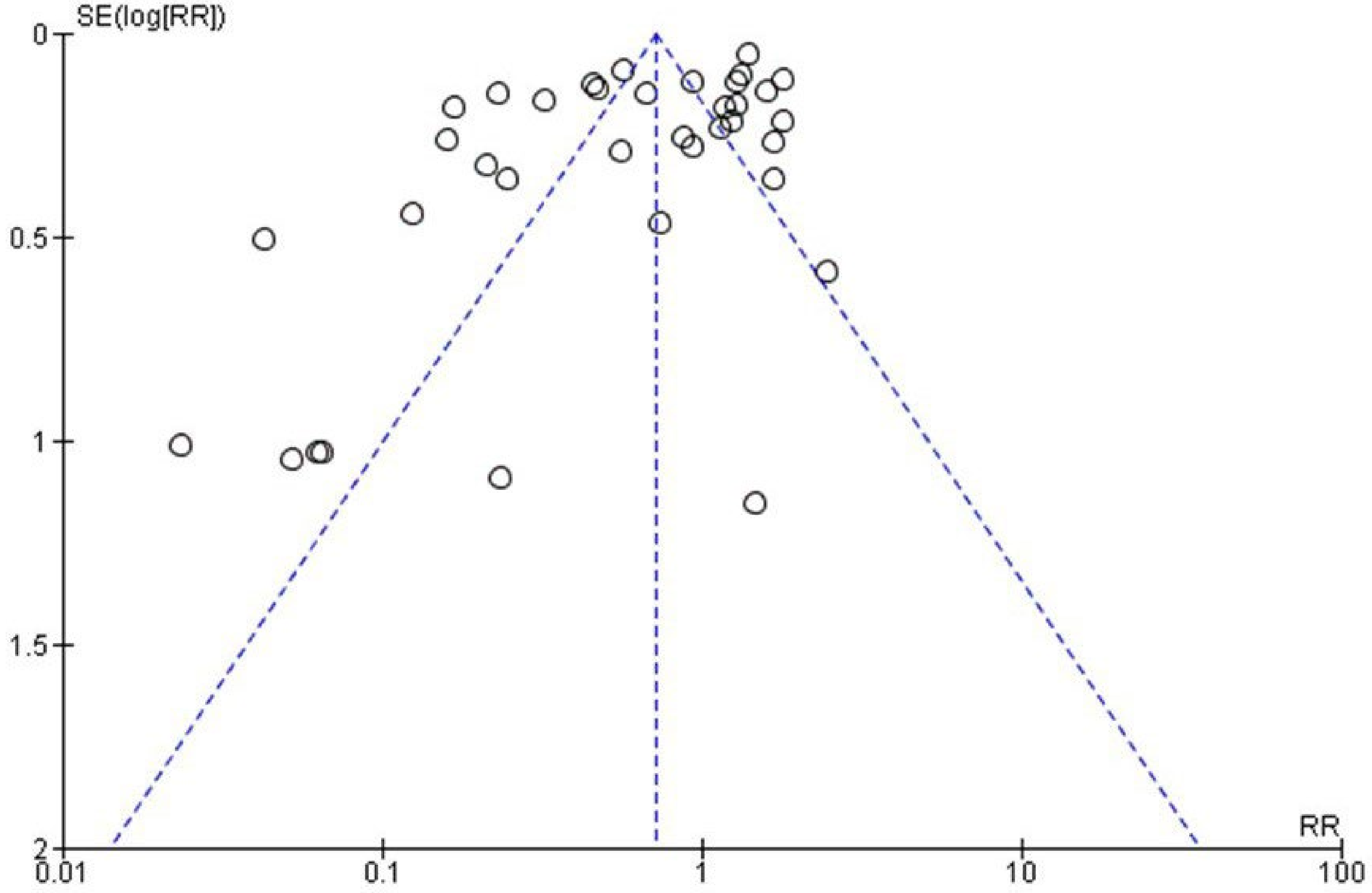

A. Funnel plots for trials included in overall analysis.

© 2022 Farooq MZ et al. *JAMA Network Open.*

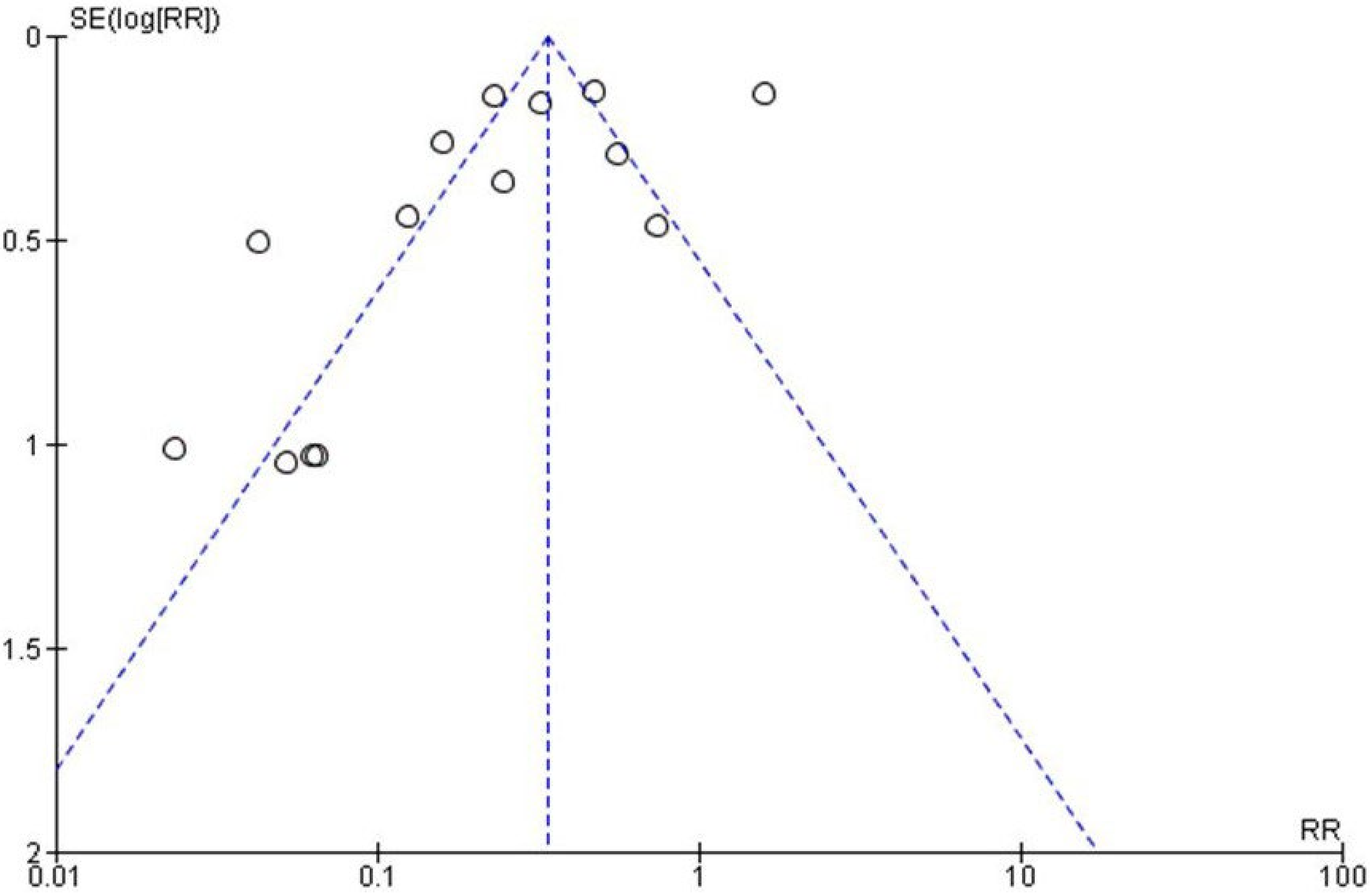

B. Funnel plots for trials included in the subgroup analysis of immunotherapy versus chemotherapy.

© 2022 Farooq MZ et al. *JAMA Network Open.*

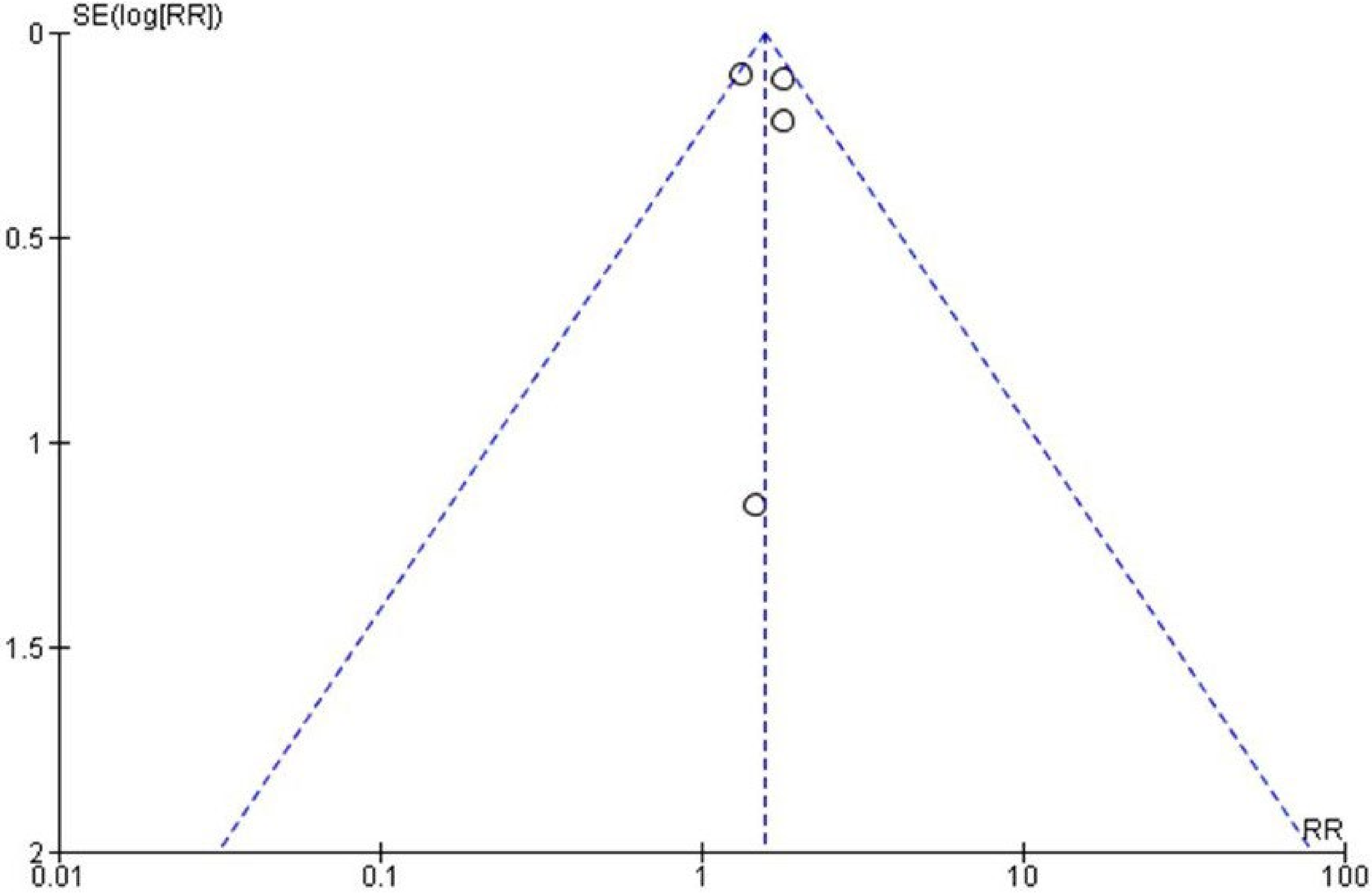

C. Funnel plots for trials included in the subgroup analysis of immunotherapy versus placebo

© 2022 Farooq MZ et al. *JAMA Network Open.*

eFigure 2. Overall Analysis of Incidence of Adverse Events Between Checkpoint Inhibitor and Control Arm

Study or Subgroup	Immunotherapy Events	Immunotherapy Total	Control Events	Control Total	Weight	Risk Ratio M-H, Random, 95% CI	Year
Reck (III)	57	84	32	44	6.0%	0.93 [0.74, 1.18]	2012
Kwon	5	393	8	396	4.9%	0.63 [0.21, 1.91]	2014
Borghaei	9	287	25	268	5.5%	0.34 [0.16, 0.71]	2015
Fehrenbacher	1	142	15	135	3.3%	0.06 [0.01, 0.47]	2016
Ferris	1	236	7	111	3.2%	0.07 [0.01, 0.54]	2016
Herbst	11	782	52	309	5.6%	0.08 [0.04, 0.16]	2016
Reck (II)	10	478	4	476	4.8%	2.49 [0.79, 7.88]	2016
Bellmunt	3	266	59	255	4.8%	0.05 [0.02, 0.15]	2017
Carbone	1	267	15	263	3.3%	0.07 [0.01, 0.49]	2017
Govindan	44	388	63	361	5.9%	0.65 [0.45, 0.93]	2017
Hamid	3	361	14	179	4.6%	0.11 [0.03, 0.36]	2017
Maio	1	380	1	189	2.4%	0.50 [0.03, 7.91]	2017
Rittmeyer	24	609	65	578	5.8%	0.35 [0.22, 0.55]	2017
Horn	6	198	4	196	4.6%	1.48 [0.43, 5.18]	2018
Paz-Ares	57	278	45	280	5.9%	1.28 [0.90, 1.82]	2018
Powles	3	459	50	443	4.8%	0.06 [0.02, 0.18]	2018
Shitara	1	294	40	276	3.4%	0.02 [0.00, 0.17]	2018
Bang	1	57	0	45	2.0%	2.38 [0.10, 57.05]	2018
Usmani	1	149	0	145	2.0%	2.92 [0.12, 71.10]	2019
West	53	473	21	232	5.8%	1.24 [0.77, 2.00]	2019
Cohen	1	246	11	234	3.3%	0.09 [0.01, 0.66]	2019
Eng	5	269	1	80	3.1%	1.49 [0.18, 12.54]	2019
Mok	4	636	90	615	5.1%	0.04 [0.02, 0.12]	2019
**Total (95% CI)**		**7732**		**6110**	**100.0%**	**0.30 [0.17, 0.51]**	
Total events	302		622				

Heterogeneity: Tau² = 1.26; Chi² = 242.08, df = 22 (P < 0.00001); I² = 91%

Test for overall effect: Z = 4.36 (P < 0.0001)

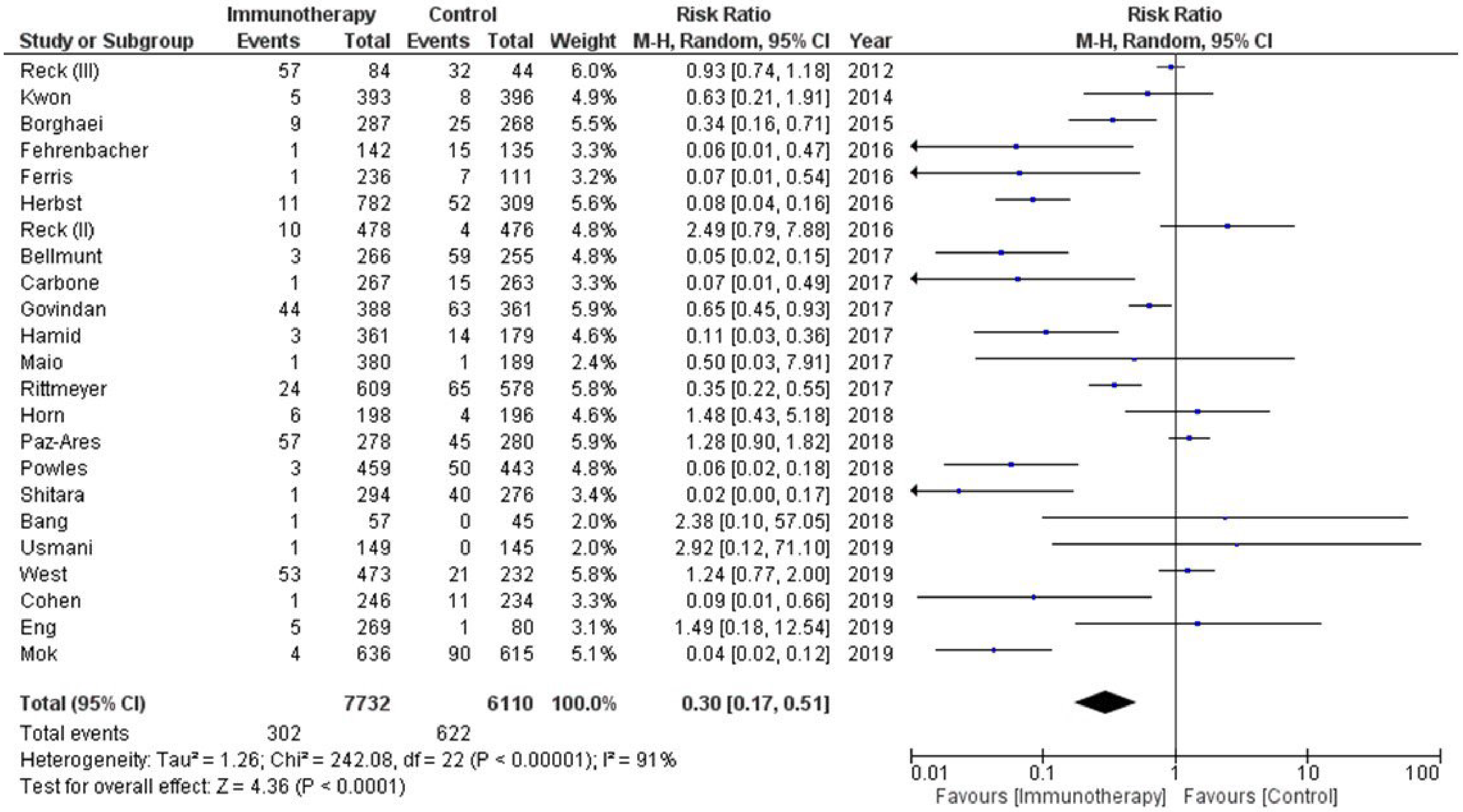

A. Peripheral neuropathy

© 2022 Farooq MZ et al. *JAMA Network Open.*

Study or Subgroup	Immunotherapy Events	Immunotherapy Total	Control Events	Control Total	Weight	Risk Ratio M-H, Random, 95% CI	Year
Hodi	84	511	19	132	7.5%	1.14 [0.72, 1.81]	2010
Robert	40	247	33	251	8.0%	1.23 [0.80, 1.89]	2011
Kwon	39	393	32	396	7.7%	1.23 [0.79, 1.92]	2014
Borghaei	29	287	2	268	1.5%	13.54 [3.26, 56.20]	2015
Eggermont (II)	152	471	86	474	11.4%	1.78 [1.41, 2.24]	2015
Herbst	14	782	2	309	1.4%	2.77 [0.63, 12.10]	2016
Maio	20	380	6	189	3.3%	1.66 [0.68, 4.06]	2017
Bellmunt	13	266	13	255	4.3%	0.96 [0.45, 2.03]	2017
Horn	42	198	38	196	8.6%	1.09 [0.74, 1.62]	2018
Barlesi	7	393	9	365	2.9%	0.72 [0.27, 1.92]	2018
Gandhi	57	405	23	202	7.6%	1.24 [0.79, 1.95]	2018
Mateos	15	120	5	121	2.9%	3.02 [1.14, 8.06]	2019
Rini (I)	48	451	30	446	7.9%	1.58 [1.02, 2.45]	2019
Rini (II)	68	429	69	425	10.1%	0.98 [0.72, 1.33]	2019
West	77	473	23	232	7.8%	1.64 [1.06, 2.55]	2019
Cohen	5	246	6	234	2.1%	0.79 [0.25, 2.56]	2019
Eng	26	269	10	80	4.8%	0.77 [0.39, 1.53]	2019
**Total (95% CI)**		**6321**		**4575**	**100.0%**	**1.32 [1.10, 1.59]**	
Total events	736		406				

Heterogeneity: $Tau^2 = 0.07$; $Chi^2 = 32.88$, df = 16 (P = 0.008); $I^2 = 51\%$
Test for overall effect: Z = 2.92 (P = 0.003)

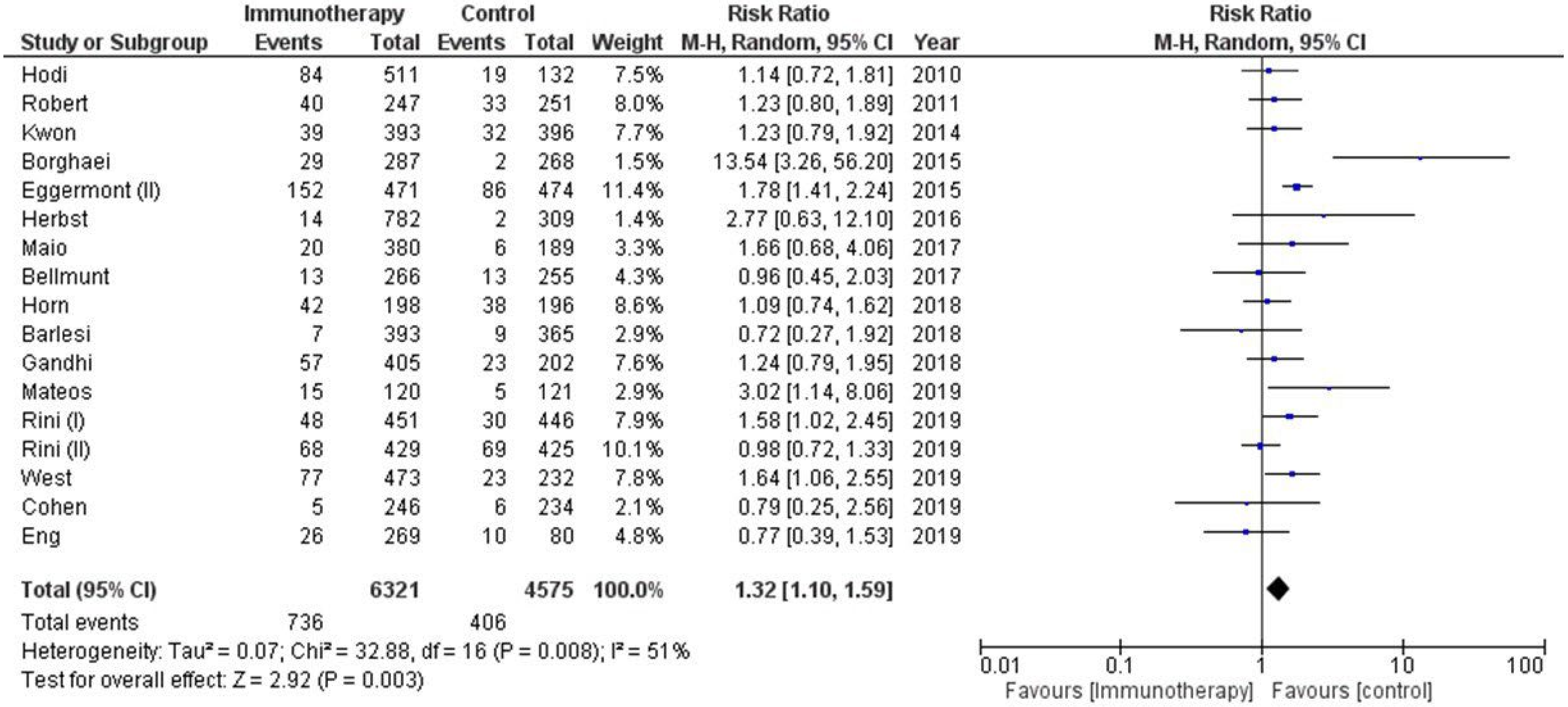

B. Headache

© 2022 Farooq MZ et al. *JAMA Network Open.*

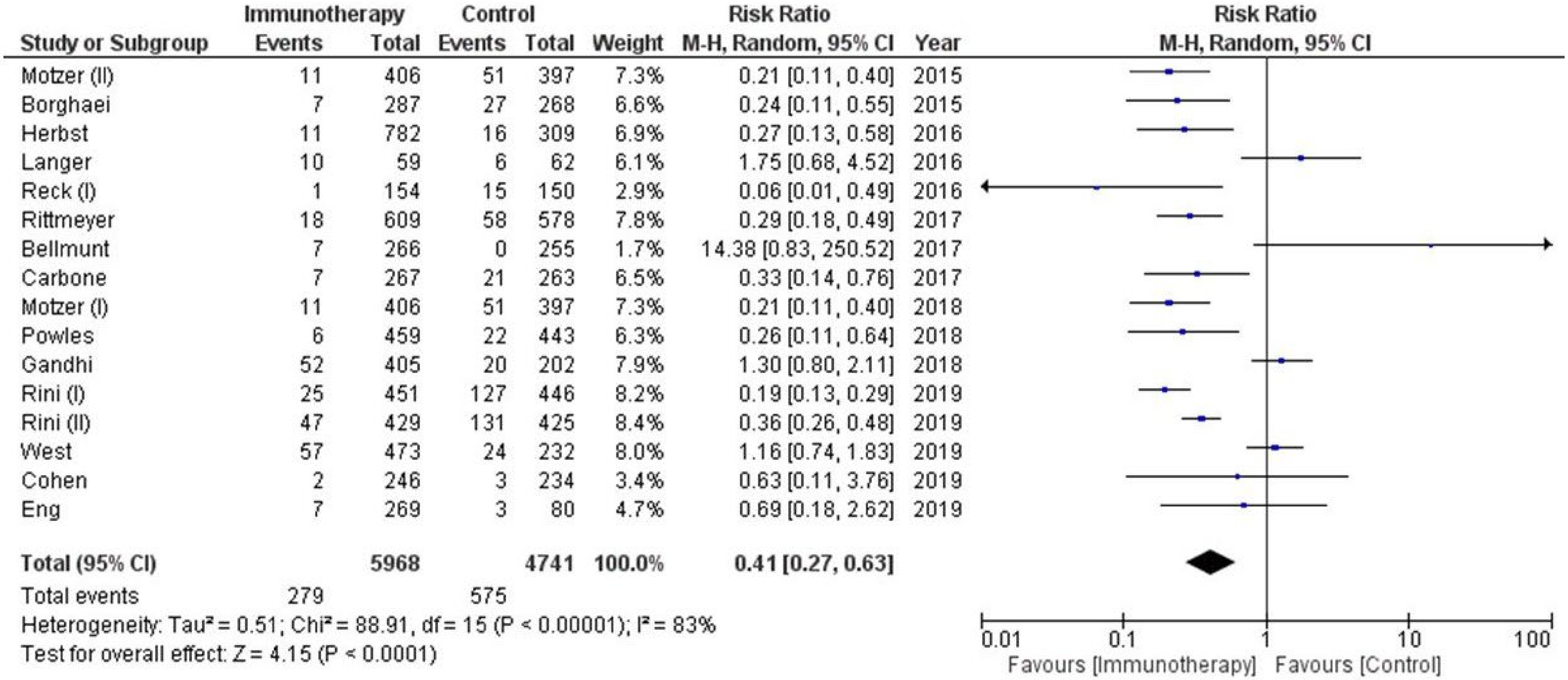

Study or Subgroup	Immunotherapy Events	Immunotherapy Total	Control Events	Control Total	Weight	Risk Ratio M-H, Random, 95% CI	Year
Motzer (II)	11	406	51	397	7.3%	0.21 [0.11, 0.40]	2015
Borghaei	7	287	27	268	6.6%	0.24 [0.11, 0.55]	2015
Herbst	11	782	16	309	6.9%	0.27 [0.13, 0.58]	2016
Langer	10	59	6	62	6.1%	1.75 [0.68, 4.52]	2016
Reck (I)	1	154	15	150	2.9%	0.06 [0.01, 0.49]	2016
Rittmeyer	18	609	58	578	7.8%	0.29 [0.18, 0.49]	2017
Bellmunt	7	266	0	255	1.7%	14.38 [0.83, 250.52]	2017
Carbone	7	267	21	263	6.5%	0.33 [0.14, 0.76]	2017
Motzer (I)	11	406	51	397	7.3%	0.21 [0.11, 0.40]	2018
Powles	6	459	22	443	6.3%	0.26 [0.11, 0.64]	2018
Gandhi	52	405	20	202	7.9%	1.30 [0.80, 2.11]	2018
Rini (I)	25	451	127	446	8.2%	0.19 [0.13, 0.29]	2019
Rini (II)	47	429	131	425	8.4%	0.36 [0.26, 0.48]	2019
West	57	473	24	232	8.0%	1.16 [0.74, 1.83]	2019
Cohen	2	246	3	234	3.4%	0.63 [0.11, 3.76]	2019
Eng	7	269	3	80	4.7%	0.69 [0.18, 2.62]	2019
**Total (95% CI)**		**5968**		**4741**	**100.0%**	**0.41 [0.27, 0.63]**	
Total events	279		575				

Heterogeneity: Tau² = 0.51; Chi² = 88.91, df = 15 (P < 0.00001); I² = 83%

Test for overall effect: Z = 4.15 (P < 0.0001)

C. Dysgeusia

© 2022 Farooq MZ et al. *JAMA Network Open.*

Study or Subgroup	Immunotherapy Events	Immunotherapy Total	Control Events	Control Total	Weight	Risk Ratio M-H, Random, 95% CI	Year
Kwon	23	393	18	396	13.2%	1.29 [0.71, 2.35]	2014
Borghaei	25	287	1	268	3.7%	23.34 [3.19, 171.09]	2015
Herbst	11	782	5	309	8.7%	0.87 [0.30, 2.48]	2016
Langer	6	59	4	62	7.4%	1.58 [0.47, 5.31]	2016
Ferris	0	236	2	111	1.8%	0.09 [0.00, 1.95]	2016
Bellmunt	15	266	19	255	12.6%	0.76 [0.39, 1.46]	2017
Maio	18	380	3	189	7.4%	2.98 [0.89, 10.00]	2017
Gandhi	52	405	20	202	14.5%	1.30 [0.80, 2.11]	2018
Cohen	0	246	2	234	1.8%	0.19 [0.01, 3.94]	2019
Mateos	15	120	30	121	13.6%	0.50 [0.29, 0.89]	2019
West	76	473	25	232	15.2%	1.49 [0.98, 2.28]	2019
**Total (95% CI)**		**3647**		**2379**	**100.0%**	**1.16 [0.75, 1.79]**	
Total events	241		129				

Heterogeneity: Tau² = 0.27; Chi² = 27.99, df = 10 (P = 0.002); I² = 64%

Test for overall effect: Z = 0.68 (P = 0.50)

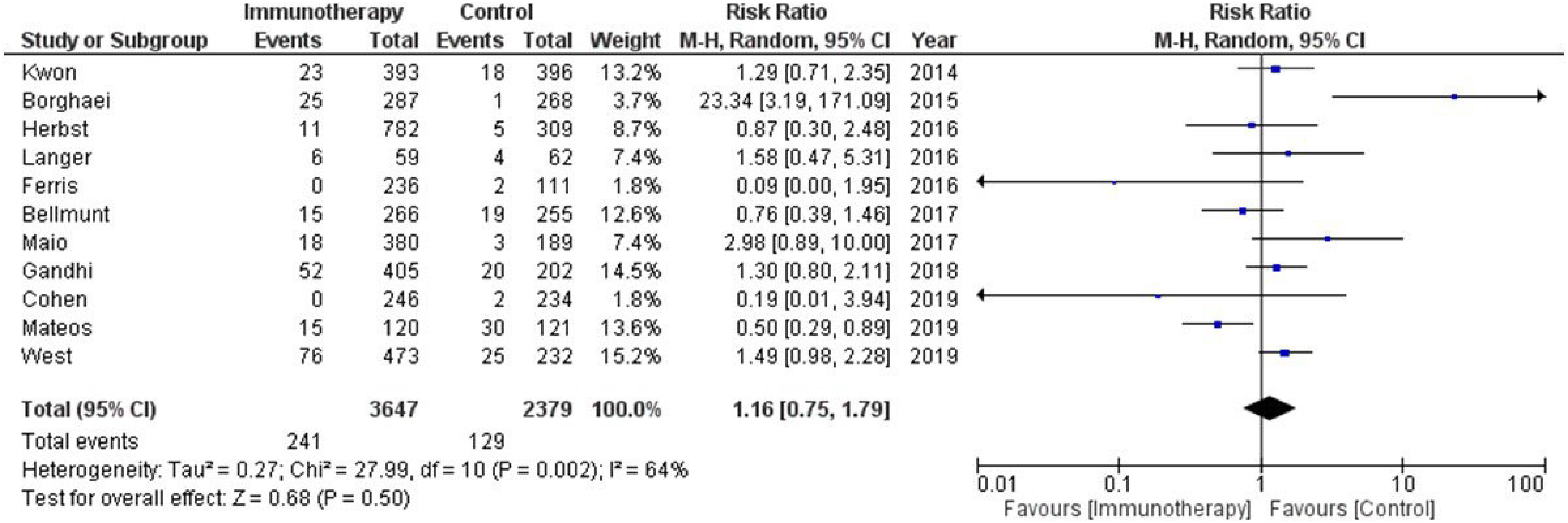

D. Dizziness

© 2022 Farooq MZ et al. *JAMA Network Open.*

Study or Subgroup	Immunotherapy Events	Immunotherapy Total	Control Events	Control Total	Weight	Risk Ratio M-H, Random, 95% CI	Year
Kwon	11	393	10	396	11.9%	1.11 [0.48, 2.58]	2014
Borghaei	5	287	0	268	4.1%	10.27 [0.57, 184.92]	2015
Herbst	9	782	22	309	12.3%	0.16 [0.08, 0.35]	2016
Hamid	3	361	10	179	9.7%	0.15 [0.04, 0.53]	2017
Maio	15	380	2	189	8.8%	3.73 [0.86, 16.14]	2017
Govindan	17	388	25	361	13.0%	0.63 [0.35, 1.15]	2017
Powles	7	459	25	443	12.0%	0.27 [0.12, 0.62]	2018
West	41	473	12	232	12.9%	1.68 [0.90, 3.13]	2019
Chih-Hsin Yang	1	17	3	12	6.1%	0.24 [0.03, 2.00]	2019
Cohen	3	246	6	234	9.2%	0.48 [0.12, 1.88]	2019
**Total (95% CI)**		**3786**		**2623**	**100.0%**	**0.61 [0.31, 1.23]**	
Total events	112		115				

Heterogeneity: $Tau^2 = 0.87$; $Chi^2 = 42.57$, df = 9 (P < 0.00001); $I^2 = 79\%$

Test for overall effect: Z = 1.38 (P = 0.17)

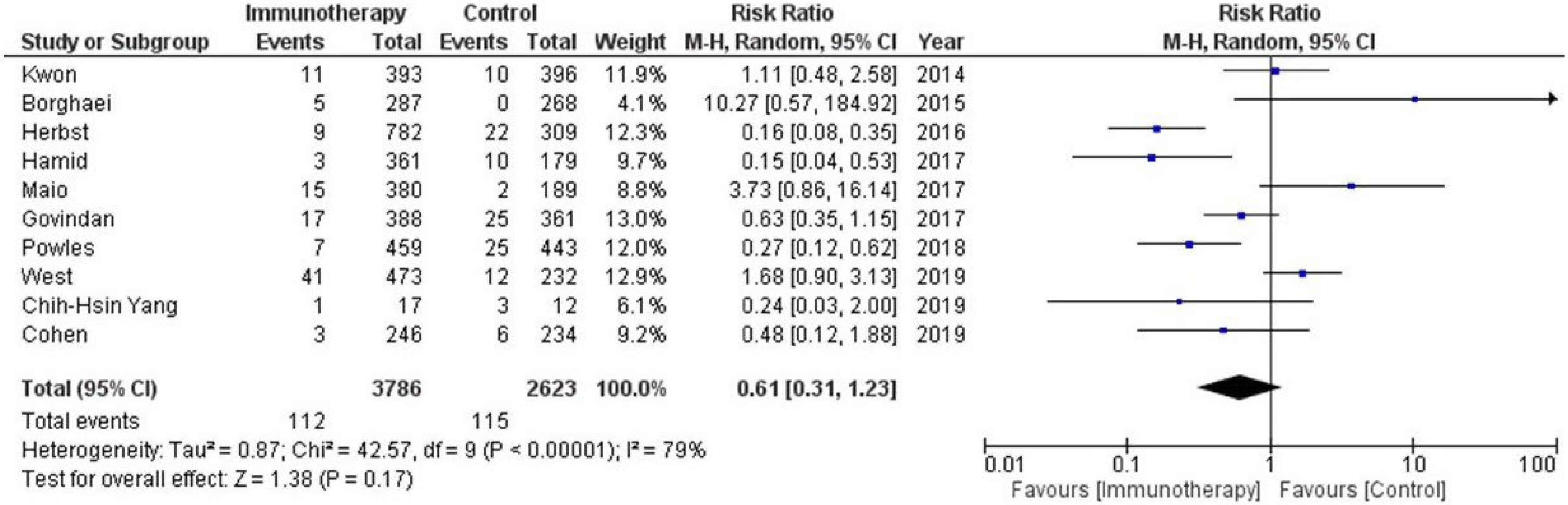

E. Paresthesia

© 2022 Farooq MZ et al. *JAMA Network Open.*

Study or Subgroup	Immunotherapy Events	Immunotherapy Total	Control Events	Control Total	Weight	Risk Ratio M-H, Random, 95% CI	Year
Kwon	31	393	24	396	57.2%	1.30 [0.78, 2.18]	2014
Carbone	1	267	0	263	1.7%	2.96 [0.12, 72.22]	2017
Maio	20	380	10	189	29.9%	0.99 [0.48, 2.08]	2017
Barlesi	0	393	1	365	1.7%	0.31 [0.01, 7.58]	2018
Cohen	1	246	0	234	1.7%	2.85 [0.12, 69.72]	2019
Eng	3	269	1	80	3.4%	0.89 [0.09, 8.46]	2019
West	20	473	1	232	4.3%	9.81 [1.32, 72.65]	2019
**Total (95% CI)**		**2421**		**1759**	**100.0%**	**1.30 [0.85, 1.97]**	
Total events	76		37				

Heterogeneity: $Tau^2 = 0.01$; $Chi^2 = 6.15$, df = 6 (P = 0.41); $I^2 = 2\%$

Test for overall effect: Z = 1.22 (P = 0.22)

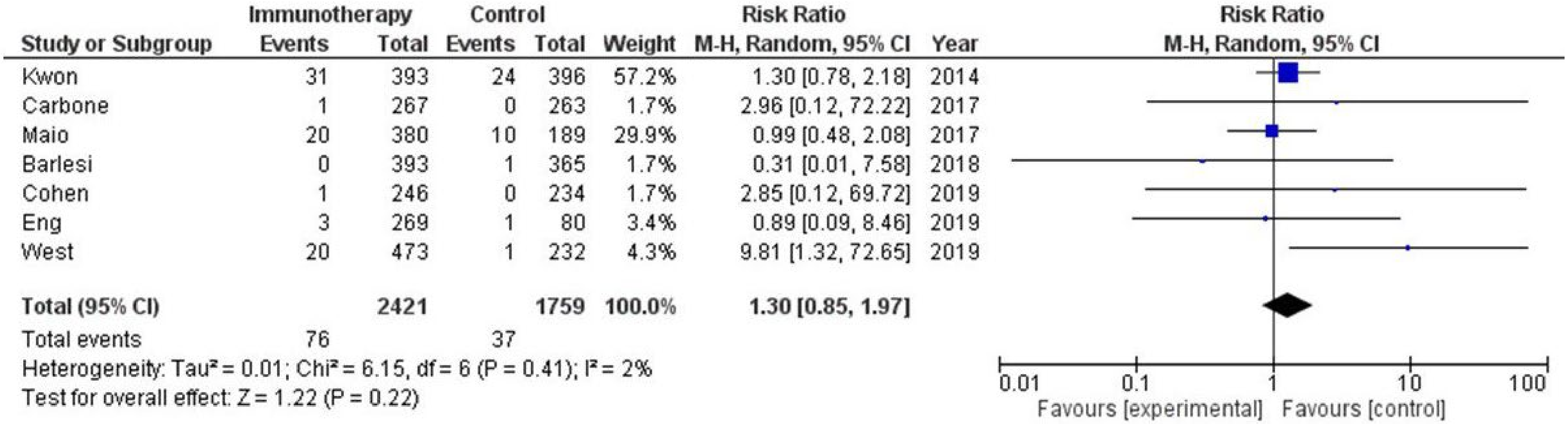

F. Altered mental status

© 2022 Farooq MZ et al. *JAMA Network Open.*

Study or Subgroup	Immunotherapy Events	Immunotherapy Total	Control Events	Control Total	Weight	Risk Ratio M-H, Random, 95% CI	Year
Kwon	31	393	0	396	5.5%	63.48 [3.90, 1033.76]	2014
Borghaei	20	287	2	268	12.4%	9.34 [2.20, 39.57]	2015
Herbst	4	782	6	309	13.9%	0.26 [0.07, 0.93]	2016
Bellmunt	16	266	19	255	19.4%	0.81 [0.42, 1.53]	2017
Usmani	19	149	22	145	20.0%	0.84 [0.48, 1.49]	2019
West	68	473	31	232	21.3%	1.08 [0.73, 1.60]	2019
Cohen	3	246	1	234	7.5%	2.85 [0.30, 27.24]	2019
**Total (95% CI)**		**2596**		**1839**	**100.0%**	**1.40 [0.66, 2.99]**	
Total events	161		81				

Heterogeneity: $Tau^2 = 0.66$; $Chi^2 = 28.96$, df = 6 (P < 0.0001); $I^2 = 79\%$

Test for overall effect: Z = 0.88 (P = 0.38)

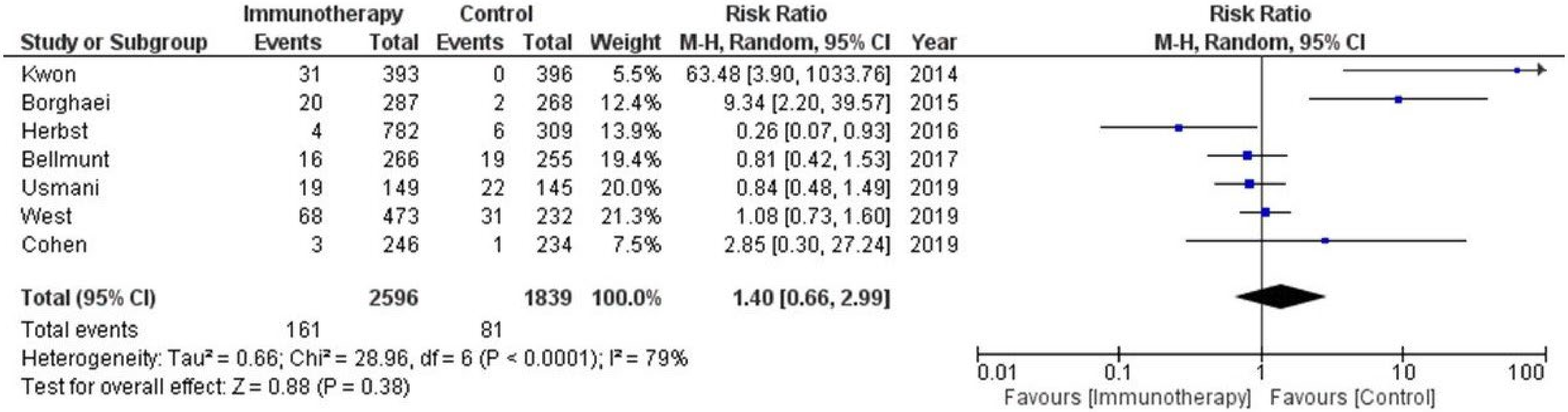

G. Insomnia

© 2022 Farooq MZ et al. *JAMA Network Open.*

eFigure 3. Subgroup Analysis of Adverse Events Between Checkpoint Inhibitors and Chemotherapy

	Immunotherapy		Chemotherapy			Risk Ratio	
Study or Subgroup	Events	Total	Events	Total	Weight	M-H, Random, 95% CI	Year
Borghaei	9	287	25	268	11.2%	0.34 [0.16, 0.71]	2015
Fehrenbacher	1	142	15	135	5.6%	0.06 [0.01, 0.47]	2016
Ferris	1	236	7	111	5.4%	0.07 [0.01, 0.54]	2016
Herbst	11	782	52	309	11.7%	0.08 [0.04, 0.16]	2016
Carbone	1	267	15	263	5.6%	0.07 [0.01, 0.49]	2017
Hamid	3	361	14	179	8.8%	0.11 [0.03, 0.36]	2017
Rittmeyer	24	609	65	578	12.4%	0.35 [0.22, 0.55]	2017
Bellmunt	3	266	59	255	9.2%	0.05 [0.02, 0.15]	2017
Shitara	1	294	40	276	5.7%	0.02 [0.00, 0.17]	2018
Powles	3	459	50	443	9.1%	0.06 [0.02, 0.18]	2018
Mok	4	636	90	615	9.9%	0.04 [0.02, 0.12]	2019
Cohen	1	246	11	234	5.5%	0.09 [0.01, 0.66]	2019
**Total (95% CI)**		**4585**		**3666**	**100.0%**	**0.09 [0.05, 0.17]**	
Total events	62		443				

Heterogeneity: Tau² = 0.77; Chi² = 43.14, df = 11 (P < 0.0001); I² = 74%
Test for overall effect: Z = 7.47 (P < 0.00001)

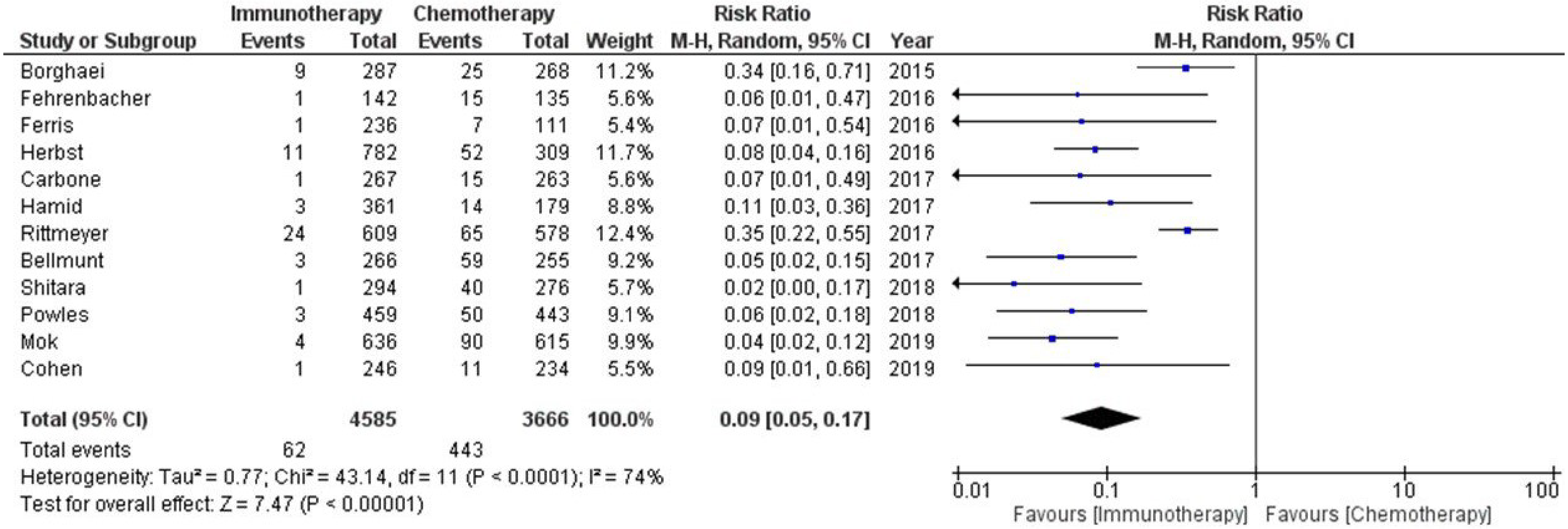

A. Peripheral neuropathy

© 2022 Farooq MZ et al. *JAMA Network Open.*

Study or Subgroup	Immunotherapy Events	Immunotherapy Total	Chemotherapy Events	Chemotherapy Total	Weight	Risk Ratio M-H, Random, 95% CI	Year
Borghaei	7	287	27	268	16.1%	0.24 [0.11, 0.55]	2015
Reck (I)	1	154	15	150	7.6%	0.06 [0.01, 0.49]	2016
Herbst	11	782	16	309	16.6%	0.27 [0.13, 0.58]	2016
Langer	10	59	6	62	14.9%	1.75 [0.68, 4.52]	2016
Bellmunt	7	266	0	255	4.6%	14.38 [0.83, 250.52]	2017
Carbone	7	267	21	263	15.9%	0.33 [0.14, 0.76]	2017
Rittmeyer	609	18	578	58		Not estimable	2017
Powles	6	459	22	443	15.4%	0.26 [0.11, 0.64]	2018
Cohen	2	246	3	234	8.8%	0.63 [0.11, 3.76]	2019
**Total (95% CI)**		**2538**		**2042**	**100.0%**	**0.42 [0.21, 0.85]**	
Total events	660		688				

Heterogeneity: Tau² = 0.62; Chi² = 22.22, df = 7 (P = 0.002); I² = 68%
Test for overall effect: Z = 2.43 (P = 0.02)

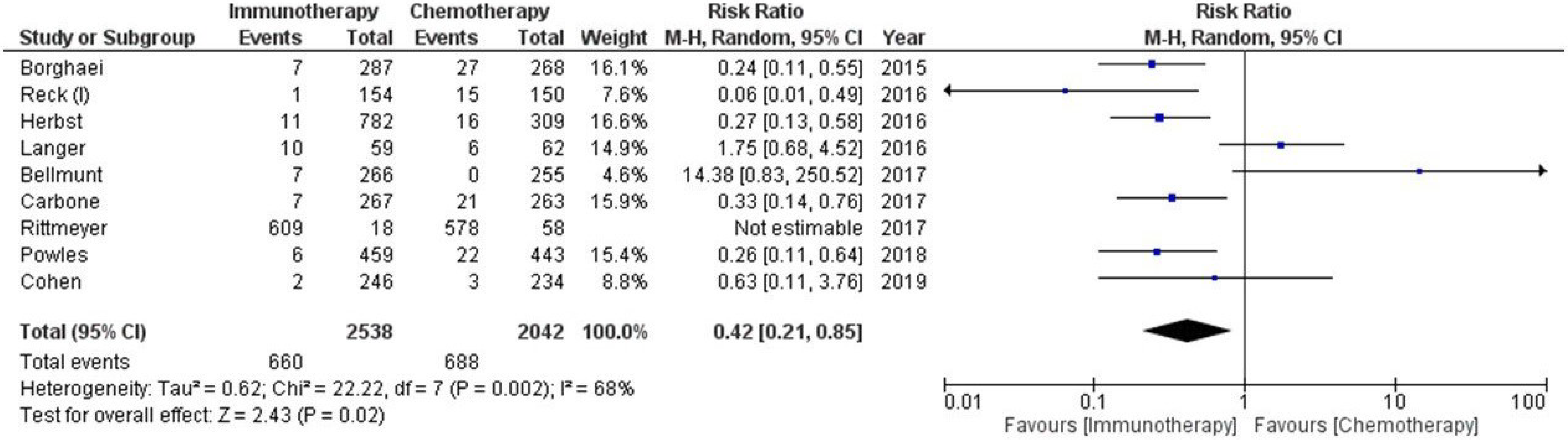

B. Dysgeusia

© 2022 Farooq MZ et al. *JAMA Network Open.*

Study or Subgroup	Immunotherapy Events	Immunotherapy Total	Chemotherapy Events	Chemotherapy Total	Weight	Risk Ratio M-H, Random, 95% CI	Year
Borghaei	5	287	0	268	6.6%	10.27 [0.57, 184.92]	2015
Herbst	9	782	22	309	28.3%	0.16 [0.08, 0.35]	2016
Hamid	3	361	10	179	19.6%	0.15 [0.04, 0.53]	2017
Powles	7	459	25	443	27.1%	0.27 [0.12, 0.62]	2018
Cohen	3	246	6	234	18.3%	0.48 [0.12, 1.88]	2019
**Total (95% CI)**		**2135**		**1433**	**100.0%**	**0.29 [0.13, 0.67]**	
Total events	27		63				

Heterogeneity: Tau² = 0.47; Chi² = 9.64, df = 4 (P = 0.05); I² = 58%
Test for overall effect: Z = 2.93 (P = 0.003)

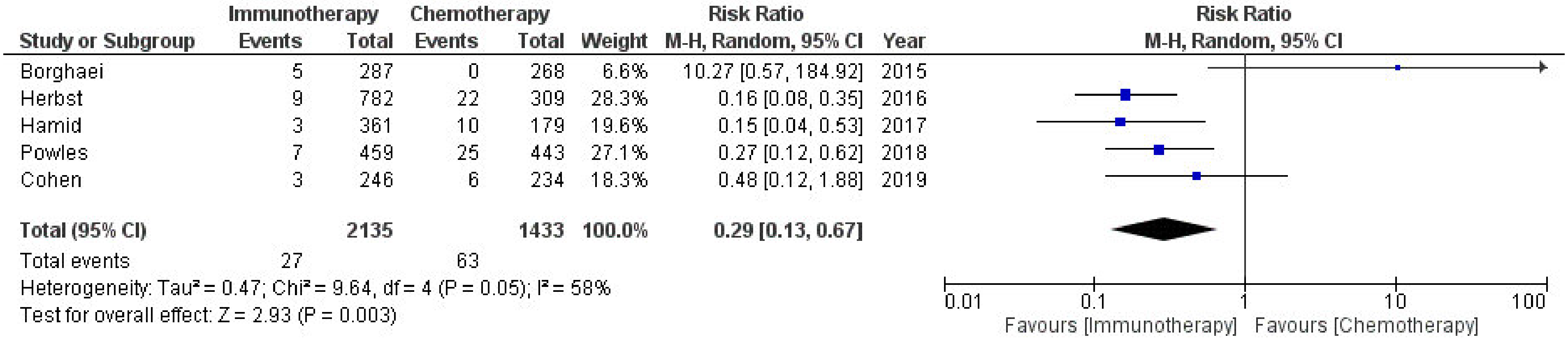

C. Paresthesia

© 2022 Farooq MZ et al. *JAMA Network Open.*

Study or Subgroup	Immunotherapy Events	Immunotherapy Total	Chemotherapy Events	Chemotherapy Total	Weight	Risk Ratio M-H, Random, 95% CI	Year
Borghaei	29	287	2	268	17.6%	13.54 [3.26, 56.20]	2015
Herbst	14	782	2	309	17.1%	2.77 [0.63, 12.10]	2016
Bellmunt	13	266	13	255	23.8%	0.96 [0.45, 2.03]	2017
Barlesi	7	393	9	365	21.7%	0.72 [0.27, 1.92]	2018
Cohen	5	246	6	234	19.9%	0.79 [0.25, 2.56]	2019
**Total (95% CI)**		**1974**		**1431**	**100.0%**	**1.66 [0.61, 4.46]**	
Total events	68		32				

Heterogeneity: Tau² = 0.93; Chi² = 15.92, df = 4 (P = 0.003); I² = 75%
Test for overall effect: Z = 1.00 (P = 0.32)

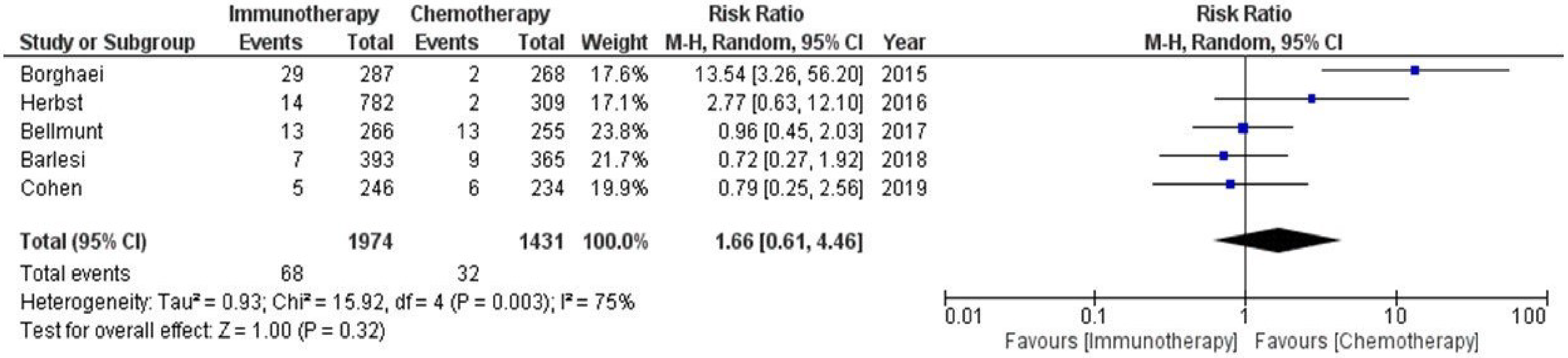

D. Headache

© 2022 Farooq MZ et al. *JAMA Network Open.*

Study or Subgroup	Immunotherapy Events	Immunotherapy Total	Chemotherapy Events	Chemotherapy Total	Weight	Risk Ratio M-H, Random, 95% CI	Year
Borghaei	20	287	2	268	24.7%	9.34 [2.20, 39.57]	2015
Herbst	4	782	6	309	26.3%	0.26 [0.07, 0.93]	2016
Bellmunt	16	266	19	255	30.7%	0.81 [0.42, 1.53]	2017
Cohen	3	246	1	234	18.3%	2.85 [0.30, 27.24]	2019
**Total (95% CI)**		**1581**		**1066**	**100.0%**	**1.39 [0.32, 5.97]**	
Total events	43		28				

Heterogeneity: Tau² = 1.69; Chi² = 15.87, df = 3 (P = 0.001); I² = 81%

Test for overall effect: Z = 0.44 (P = 0.66)

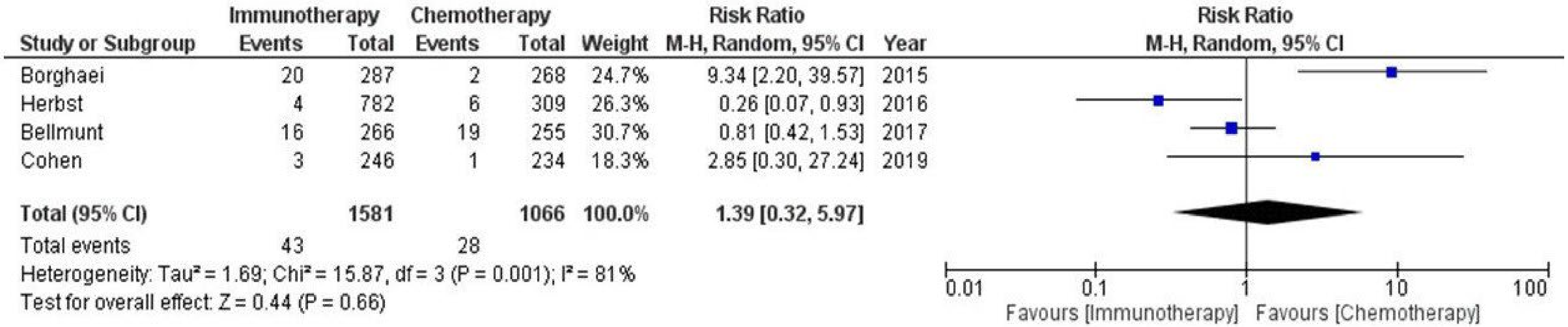

E. Insomnia

© 2022 Farooq MZ et al. *JAMA Network Open.*

Study or Subgroup	Immunotherapy Events	Immunotherapy Total	Chemotherapy Events	Chemotherapy Total	Weight	Risk Ratio M-H, Random, 95% CI	Year
Borghaei	25	287	1	268	19.0%	23.34 [3.19, 171.09]	2015
Ferris	0	236	2	111	12.6%	0.09 [0.00, 1.95]	2016
Herbst	11	782	5	309	26.6%	0.87 [0.30, 2.48]	2016
Bellmunt	15	266	19	255	29.3%	0.76 [0.39, 1.46]	2017
Cohen	0	246	2	234	12.5%	0.19 [0.01, 3.94]	2019
**Total (95% CI)**		**1817**		**1177**	**100.0%**	**0.98 [0.24, 3.91]**	
Total events	51		29				

Heterogeneity: Tau² = 1.60; Chi² = 16.26, df = 4 (P = 0.003); I² = 75%
Test for overall effect: Z = 0.03 (P = 0.97)

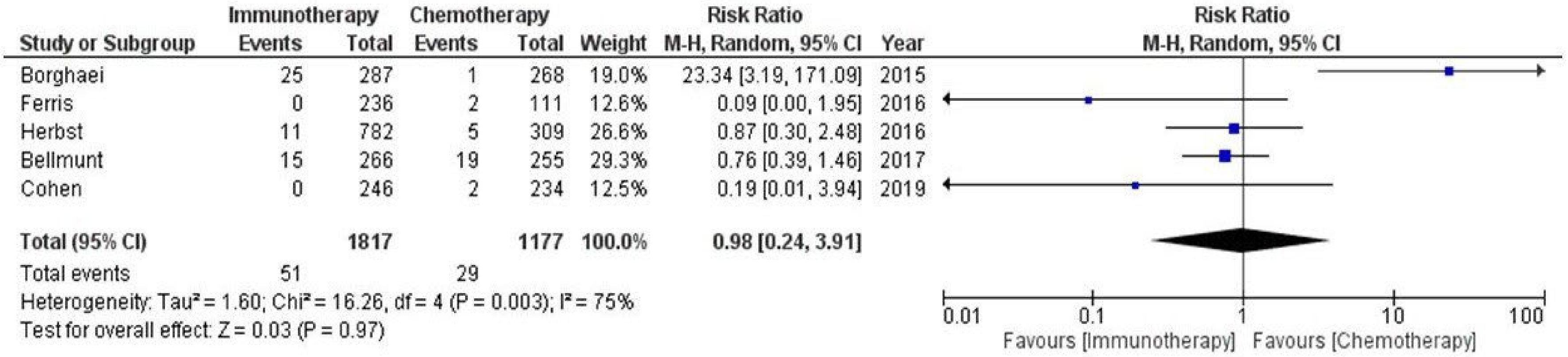

F. Dizziness

© 2022 Farooq MZ et al. *JAMA Network Open.*

eFigure 4. Subgroup Analysis of Headache Between Checkpoint Inhibitors and Placebo

	Immunotherapy		Placebo			Risk Ratio	
**Study or Subgroup**	**Events**	**Total**	**Events**	**Total**	**Weight**	**M-H, Random, 95% CI**	**Year**
Kwon	39	393	32	396	21.9%	1.23 [0.79, 1.92]	2014
Eggermont (II)	152	471	86	474	72.5%	1.78 [1.41, 2.24]	2015
Maio	20	380	6	189	5.6%	1.66 [0.68, 4.06]	2017
**Total (95% CI)**		**1244**		**1059**	**100.0%**	**1.63 [1.32, 2.02]**	
Total events	211		124				

Heterogeneity: Tau² = 0.00; Chi² = 2.09, df = 2 (P = 0.35); I² = 4%

Test for overall effect: Z = 4.50 (P < 0.00001)

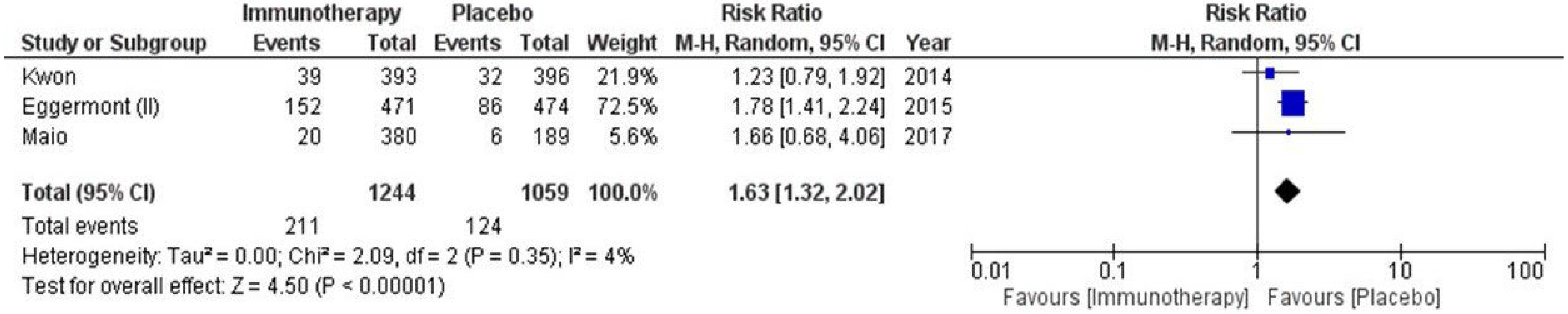

© 2022 Farooq MZ et al. *JAMA Network Open.*

eFigure 5. Overall Risk of Neurologic Adverse Events Excluding Incidence of Specific Event Between Checkpoint Inhibitors and Control Arm

	Immunotherapy		Control			Risk Ratio	
Study or Subgroup	Events	Total	Events	Total	Weight	M-H, Random, 95% CI	Year
Hodi	84	511	19	132	3.8%	1.14 [0.72, 1.81]	2010
Robert	40	247	33	251	3.8%	1.23 [0.80, 1.89]	2011
Reck (III)	0	84	0	44		Not estimable	2012
Kwon	145	393	104	396	4.1%	1.40 [1.14, 1.73]	2014
Borghaei	88	287	32	268	3.9%	2.57 [1.78, 3.71]	2015
Eggermont (II)	153	471	86	474	4.1%	1.79 [1.42, 2.26]	2015
Motzer (II)	11	406	51	397	3.4%	0.21 [0.11, 0.40]	2015
Fehrenbacher	0	142	0	135		Not estimable	2016
Ferris	0	236	2	111	0.7%	0.09 [0.00, 1.95]	2016
Herbst	49	782	51	309	3.9%	0.38 [0.26, 0.55]	2016
Langer	16	59	10	62	3.3%	1.68 [0.83, 3.40]	2016
Reck (I)	1	154	15	150	1.3%	0.06 [0.01, 0.49]	2016
Reck (II)	0	478	0	476		Not estimable	2016
Bang (II)	0	57	0	45		Not estimable	2017
Antonia	3	475	1	234	1.1%	1.48 [0.15, 14.13]	2017
Bellmunt	51	266	51	255	4.0%	0.96 [0.68, 1.36]	2017
Carbone	8	267	21	263	3.1%	0.38 [0.17, 0.83]	2017
Govindan	19	388	25	361	3.6%	0.71 [0.40, 1.26]	2017
Hamid	3	361	10	179	2.2%	0.15 [0.04, 0.53]	2017
Maio	82	380	22	189	3.8%	1.85 [1.20, 2.87]	2017
Rittmeyer	18	609	58	578	3.7%	0.29 [0.18, 0.49]	2017
Bang (I)	0	184	0	177		Not estimable	2018
Barlesi	8	393	10	365	2.8%	0.74 [0.30, 1.86]	2018
Eggermont (I)	0	509	0	502		Not estimable	2018
Gandhi	161	405	63	202	4.1%	1.27 [1.01, 1.62]	2018
Horn	44	198	38	196	3.9%	1.15 [0.78, 1.69]	2018
Motzer (I)	31	547	180	535	4.0%	0.17 [0.12, 0.24]	2018
Paz-Ares	0	278	0	280		Not estimable	2018
Powles	13	459	47	443	3.5%	0.27 [0.15, 0.49]	2018
Shitara	0	294	0	276		Not estimable	2018
Usmani	20	149	22	145	3.6%	0.88 [0.50, 1.55]	2019
West	362	473	125	232	4.2%	1.42 [1.25, 1.62]	2019
Chih-Hsin Yang	1	17	3	12	1.1%	0.24 [0.03, 2.00]	2019
Cohen	16	246	18	234	3.4%	0.85 [0.44, 1.62]	2019
Eng	42	269	15	80	3.6%	0.83 [0.49, 1.42]	2019
Mateos	30	120	18	121	3.7%	1.68 [0.99, 2.85]	2019
Mok	0	636	0	615		Not estimable	2019
Rini (I)	73	451	157	446	4.1%	0.46 [0.36, 0.59]	2019
Rini (II)	115	429	200	425	4.2%	0.57 [0.47, 0.69]	2019
**Total (95% CI)**		**13110**		**10595**	**100.0%**	**0.74 [0.56, 0.97]**	
Total events	1687		1487				

Heterogeneity: $Tau^2 = 0.44$; $Chi^2 = 404.27$, df = 29 ($P < 0.00001$); $I^2 = 93\%$
Test for overall effect: Z = 2.22 (P = 0.03)

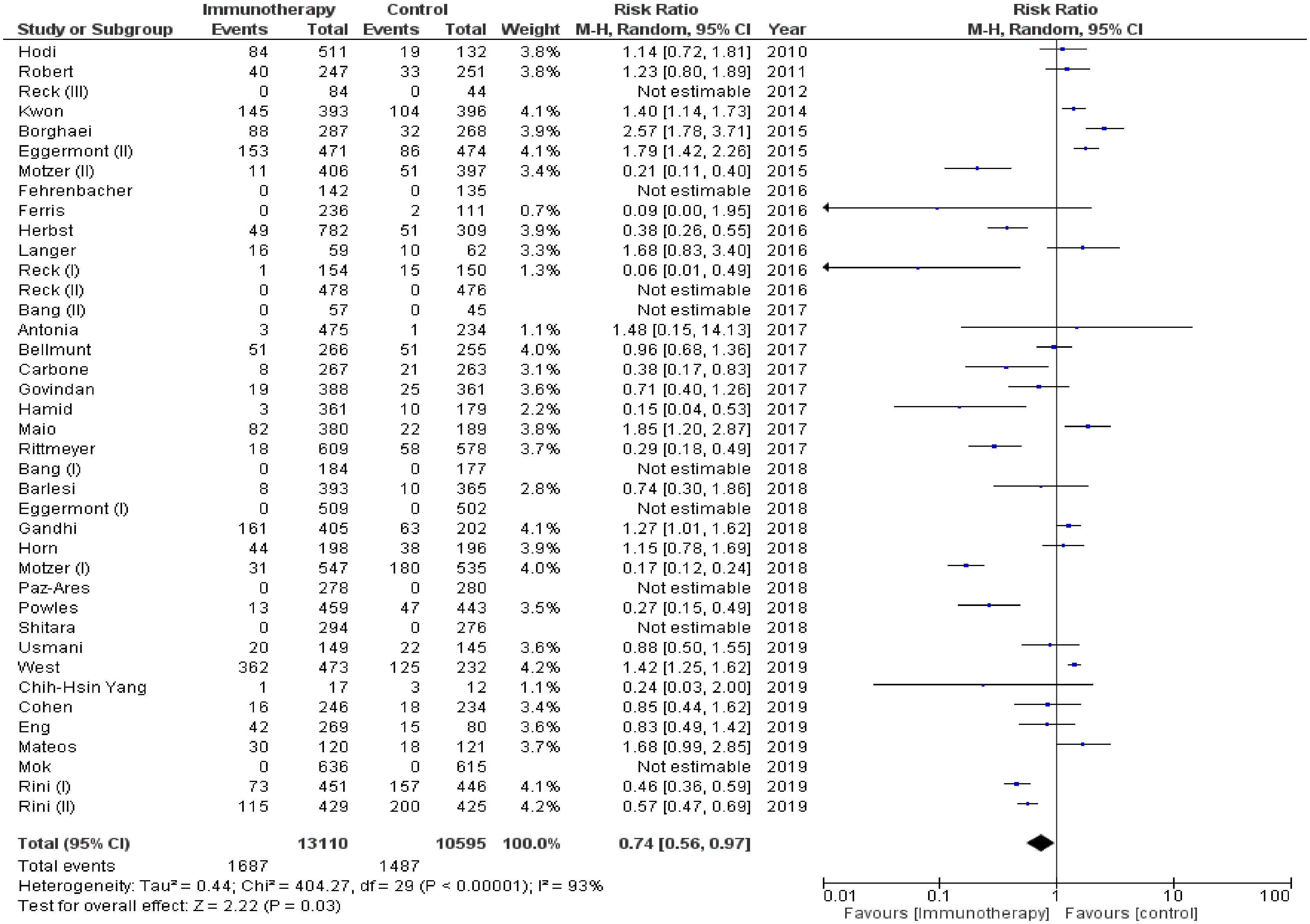

A. Excluding the Incidence of Peripheral Neuropathy

© 2022 Farooq MZ et al. *JAMA Network Open.*

Study or Subgroup	Immunotherapy Events	Immunotherapy Total	Chemotherapy Events	Chemotherapy Total	Weight	Risk Ratio M-H, Random, 95% CI	Year
Borghaei	88	287	31	268	11.3%	2.65 [1.82, 3.85]	2015
Fehrenbacher	0	142	0	135		Not estimable	2016
Ferris	0	236	2	111	2.9%	0.09 [0.00, 1.95]	2016
Herbst	49	782	51	309	11.3%	0.38 [0.26, 0.55]	2016
Reck (I)	1	154	15	150	5.0%	0.06 [0.01, 0.49]	2016
Bellmunt	51	266	51	255	11.3%	0.96 [0.68, 1.36]	2017
Carbone	8	267	21	263	9.7%	0.38 [0.17, 0.83]	2017
Hamid	3	361	10	179	7.6%	0.15 [0.04, 0.53]	2017
Rittmeyer	18	609	58	578	10.8%	0.29 [0.18, 0.49]	2017
Barlesi	8	353	10	365	9.2%	0.83 [0.33, 2.07]	2018
Powles	13	459	47	453	10.5%	0.27 [0.15, 0.50]	2018
Shitara	0	294	0	276		Not estimable	2018
Bang (I)	0	184	0	187		Not estimable	2018
Cohen	16	246	18	234	10.3%	0.85 [0.44, 1.62]	2019
Mok	0	636	0	215		Not estimable	2019
**Total (95% CI)**		**5276**		**3978**	**100.0%**	**0.47 [0.26, 0.85]**	
Total events	255		314				

Heterogeneity: Tau² = 0.78; Chi² = 98.45, df = 10 (P < 0.00001); I² = 90%
Test for overall effect: Z = 2.49 (P = 0.01)

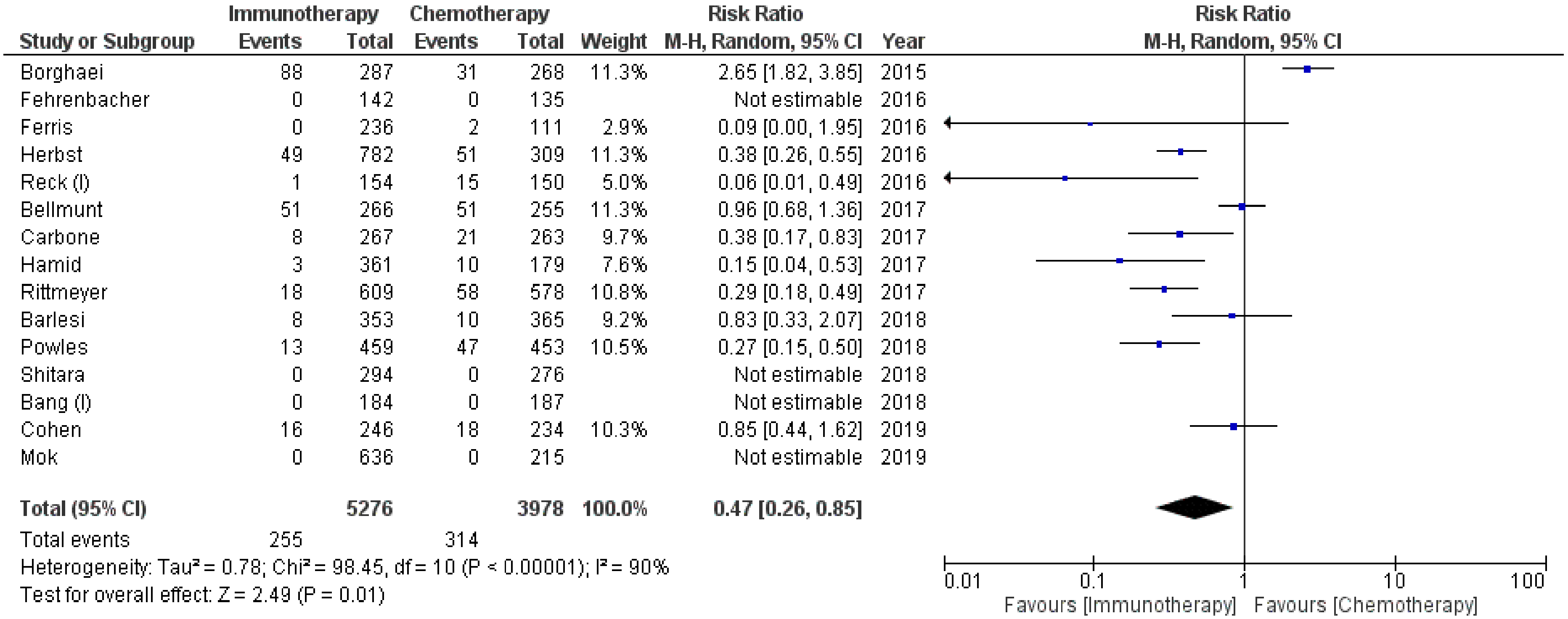

B. Excluding the Incidence of Peripheral Neuropathy

© 2022 Farooq MZ et al. *JAMA Network Open.*

eTable 1. Quality Assessment of All Trials Using the Cochrane Collaborations Tool

Article	Selection Bias		Performance Bias	Detection bias	Attrition bias	Reporting Bias	Other bias	Our evaluation
	**Random Sequence Generation**	**Allocation concealment**	**Blinding of participants and personnel**	**Blinding of outcome assessment**	**Incomplete outcome data**	**Selective Reporting**	**Anything else, ideally prespecified**	
Chih-Hsin Yang	Low risk	Low risk	Low risk	Low risk	Low risk	Low risk	Low risk	Good quality
Cohen	Low risk	Low risk	Low risk	Low risk	Low risk	Low risk	Low risk	Good quality
Eng	Low risk	Low risk	Low risk	Low risk	Low risk	Low risk	Low risk	Good quality
Mateos	Low risk	Low risk	Low risk	Low risk	Low risk	Low risk	Low risk	Good quality
Mok	Low risk	Low risk	Low risk	Low risk	Low risk	Low risk	Low risk	Good quality
Rini (1)	Low risk	Low risk	Low risk	Low risk	Low risk	Low risk	Low risk	Good quality
Rini (2)	Low risk	Low risk	Low risk	Low risk	Low risk	Low risk	Low risk	Good quality
Usmani	Low risk	Low risk	Low risk	Low risk	Low risk	Low risk	Low risk	Good quality
West	Low risk	Low risk	Low risk	Low risk	Low risk	Low risk	Low risk	Good quality
Bang (1)	Low risk	Unclear risk	Low risk	Unclear risk	Low risk	Low risk	Low risk	Fair quality
Barlesi	Low risk	Low risk	Low risk	Low risk	Low risk	Low risk	Low risk	Good quality
Eggermont	Low risk	Low risk	Low risk	Low risk	Low risk	Low risk	Low risk	Good quality
Gandhi	Low risk	Low risk	Low risk	Low risk	Low risk	Low risk	Low risk	Good quality
Horn	Low risk	Low risk	Low risk	Low risk	Low risk	Low risk	Low risk	Good quality
Motzer	Low risk	Low risk	Low risk	Low risk	Low risk	Low risk	Low risk	Good quality
Paz-Ares	Low risk	Low risk	Low risk	Low risk	Low risk	Low risk	Low risk	Good quality
Powles	Low risk	Low risk	Low risk	Low risk	Low risk	Low risk	Low risk	Good quality
Shitara	Low risk	Low risk	Low risk	Low risk	Low risk	Low risk	Low risk	Good quality
Antonia	Low risk	Low risk	Low risk	Low risk	Low risk	Low risk	Low risk	Good quality
Bang (2)	Unclear risk	Unclear risk	Low risk	Low risk	Low risk	Low risk	Low risk	Fair quality
Bellmunt	Low risk	Low risk	Low risk	Low risk	Low risk	Low risk	Low risk	Good quality
Carbone	Low risk	Unclear risk	Unclear risk	Low risk	Low risk	Low risk	Low risk	Fair quality
Govindan	Low risk	Unclear risk	Low risk	Unclear risk	Low risk	Low risk	Low risk	Fair quality
Hamid	Low risk	Low risk	Low risk	Low risk	Low risk	Low risk	Low risk	Good quality
Maio	Low risk	Low risk	Low risk	Low risk	Low risk	Low risk	Low risk	Good quality
Rittmeyer	Low risk	Low risk	Low risk	Low risk	Low risk	Low risk	Low risk	Good quality
Fehrenbacher	Low risk	Low risk	Low risk	Low risk	Low risk	Low risk	Low risk	Good quality
Ferris	Low risk	Low risk	Low risk	Low risk	Low risk	Low risk	Low risk	Good quality
Herbst	Low risk	Low risk	Low risk	Low risk	Low risk	Low risk	Low risk	Good quality
Langer	Low risk	Low risk	Low risk	Low risk	Low risk	Low risk	Low risk	Good quality
Reck (I)	Low risk	Low risk	Low risk	Low risk	Low risk	Low risk	Low risk	Good quality
Reck (II)	Low risk	Low risk	Low risk	Low risk	Low risk	Low risk	Low risk	Good quality
Borghaei	Low risk	Low risk	Low risk	Low risk	Low risk	Low risk	Low risk	Good quality

© 2022 Farooq MZ et al. *JAMA Network Open.*

Eggermont (II)	Low risk	Low risk	Low risk	Low risk	Low risk	Low risk	Low risk	Good quality
Article	Selection Bias		Performance Bias		Detection bias		Other bias	Our evaluation
	**Random Sequence Generation**	**Allocation concealment**	**Blinding of participants and personnel**	**Blinding of outcome assessment**	**Incomplete outcome data**	**Selective Reporting**	**Anything else, ideally prespecified**	
Motzer (II)	Low risk	Low risk	Low risk	Low risk	Low risk	Low risk	Low risk	Good quality
Kwon	Low risk	Low risk	Low risk	Low risk	Low risk	Low risk	Low risk	Good quality
Reck	Unclear risk	Low risk	Low risk	Unclear risk	Low risk	Low risk	Low risk	Fair quality
Robert	Low risk	Low risk	Low risk	Low risk	Low risk	Low risk	Low risk	Good quality
Hodi	Low risk	Low risk	Low risk	Low risk	Low risk	Low risk	Low risk	Good quality

© 2022 Farooq MZ et al. *JAMA Network Open.*

eTable 2. Pooled Characteristics of the Intention-to-Treat Population

**Variable**	**Control group (All drug regimens in the comparison arm)** N=10775	**Immune checkpoint inhibitor group** N=12958
**Age**	Unable to estimate	Unable to estimate
**Female sex — no. (%)**	3315 (30.8)	4283 (33.1)
**Race — no. (%)**		
White	3365 (31.2)	4501 (34.7)
African American/black	56 (0.1)	91 (0.1)
Asian	779 (7.2)	924 (7.1)
Other	50 (<0.1)	74 (0.1)
Unknown/not reported	6525 (60.1)	7368 (56.9)
**Region**		
North America	496 (4.6)	664 (5.1)
Europe	1353 (12.6)	1522 (11.8)
Asia	715 (6.6)	804 (6.2)
Latin America	192 (1.8)	205 (1.6)
Unspecified/not reported	8019 (74.4)	9763 (75.3)
**Tumor type**		
Non-small cell lung cancer	4060 (37.6)	5116 (39.5)
Renal cell carcinoma	1847 (17.1)	1846 (14.2)
Malignant melanoma	1548 (14.4)	2140 (16.5)
Small cell lung cancer	723 (6.7)	764 (5.9)
Urothelial	736 (6.8)	737 (5.7)
Gastric/gastroesophageal junction cancer	539 (5.0)	538 (4.2)
HNSCC	369 (3.4)	487 (3.8)
Prostate	400 (3.7)	399 (3.1)
**Tumor Type (continued)**		
Multiple myeloma	274 (2.5)	276 (2.1)
Malignant mesothelioma	189 (1.8)	382 (2.9)
Colorectal cancer	90 (0.1)	273 (2.1)
**ECOG performance-status score — no. (%)**		
0	2890 (26.8)	3725 (28.7)
1	4757 (44.1)	5855 (45.2)
2	23 (0.2)	22 (0.2)
Unknown/not reported/used Karnofsky performance status	3105 (28.8)	3356 (25.9)
**Smoking status — no. (%)**		
Current or former smoker	4132 (38.3)	5105 (39.4)
Never smoker	895 (8.3)	1109 (8.6)
Unknown/not reported	5748 (53.3)	6744 (52.0)

ECOG- Eastern Cooperative Oncology Group; HNSCC- Head and Neck Squamous Cell Carcinoma.

© 2022 Farooq MZ et al. *JAMA Network Open.*
